# A Genetic Strategy for Probing the Functional Diversity of Magnetosome Formation

**DOI:** 10.1371/journal.pgen.1004811

**Published:** 2015-01-08

**Authors:** Lilah Rahn-Lee, Meghan E. Byrne, Manjing Zhang, David Le Sage, David R. Glenn, Timothy Milbourne, Ronald L. Walsworth, Hojatollah Vali, Arash Komeili

**Affiliations:** 1 Department of Plant and Microbial Biology, University of California Berkeley, Berkeley, California, United States of America; 2 Department of Physics, Harvard University, Cambridge, Massachusetts, United States of America; 3 Harvard-Smithsonian Center for Astrophysics, Cambridge, Massachusetts, United States of America; 4 Center for Brain Science, Harvard University, Cambridge, Massachusetts, United States of America; 5 Facility for Electron Microscopy Research, McGill University, Montreal, Quebec, Canada; 6 Department of Anatomy and Cell Biology, McGill University, Montreal, Quebec, Canada; 7 Department of Earth and Planetary Sciences, McGill University, Montreal, Quebec, Canada; Indiana University, United States of America

## Abstract

Model genetic systems are invaluable, but limit us to understanding only a few organisms in detail, missing the variations in biological processes that are performed by related organisms. One such diverse process is the formation of magnetosome organelles by magnetotactic bacteria. Studies of model magnetotactic α-proteobacteria have demonstrated that magnetosomes are cubo-octahedral magnetite crystals that are synthesized within pre-existing membrane compartments derived from the inner membrane and orchestrated by a specific set of genes encoded within a genomic island. However, this model cannot explain all magnetosome formation, which is phenotypically and genetically diverse. For example, *Desulfovibrio magneticus* RS-1, a δ-proteobacterium for which we lack genetic tools, produces tooth-shaped magnetite crystals that may or may not be encased by a membrane with a magnetosome gene island that diverges significantly from those of the α-proteobacteria. To probe the functional diversity of magnetosome formation, we used modern sequencing technology to identify hits in RS-1 mutated with UV or chemical mutagens. We isolated and characterized mutant alleles of 10 magnetosome genes in RS-1, 7 of which are not found in the α-proteobacterial models. These findings have implications for our understanding of magnetosome formation in general and demonstrate the feasibility of applying a modern genetic approach to an organism for which classic genetic tools are not available.

## Author Summary

Model organisms are used to understand biological processes in genetic and molecular detail. However, some interesting processes lack model organisms, and many would benefit from their study in diverse organisms. Bacteria are an enormously diverse domain of life that contains only a few well-studied model systems, making much of their interesting biology inaccessible to genetic or molecular research. Here, we employ a method for performing genetic experiments on non-model organisms that takes advantage of new DNA sequencing technology to work around a lack of genetic tools. We use this method on a non-model organism, *Desulfovibrio magneticus* RS-1, a member of the magnetotactic bacteria, which construct intracellular nano-sized magnets. The current understanding of magnetotactic bacteria comes from two model organisms that differ from RS-1 in the shape of their magnets and the manner in which the particles are produced. Genetic control over the size and shape of minerals is a poorly understood process and its elucidation has implications for nanotechnological applications. Here, without the need to develop extensive genetic tools for a fastidious organism, we have extended the understanding of the molecular basis of bacterial nano-magnet production.

## Introduction

Genetic analysis historically relied on model systems that were easy to manipulate and for which genetic maps, then later full genome sequences, were available. Today many interesting organisms have their genomes sequenced, but cannot be easily manipulated due to their incompatibility with standard genetic tools. One such organism is *Desulfovibrio magneticus* RS-1 (RS-1).

RS-1 is a member of the magnetotactic bacteria, a phylogenetically diverse group of gram-negative bacteria that synthesize magnetic iron oxide (magnetite) or iron sulfide (greigite) crystals within complex genetically encoded intracellular organelles. Magnetotactic bacteria were independently discovered by Bellini in 1963 [1], [2] and Blakemore in 1975 [3]. Both investigators observed bacteria that aligned with a magnetic field. It was hypothesized that this behavior allows the bacteria, which were isolated in the northern hemisphere, to easily swim northwards to the bottom of the water column where micro-oxic or anoxic conditions exist. This behavior is due to intracellular single-domain magnetite or greigite crystals organized in one or more chains along the length of the cell [4]. Since their discovery, magnetotactic bacteria have inspired studies in bacterial cell biology [5], biomineralization [6], and nanotechnology [7].

**Figure 1:**
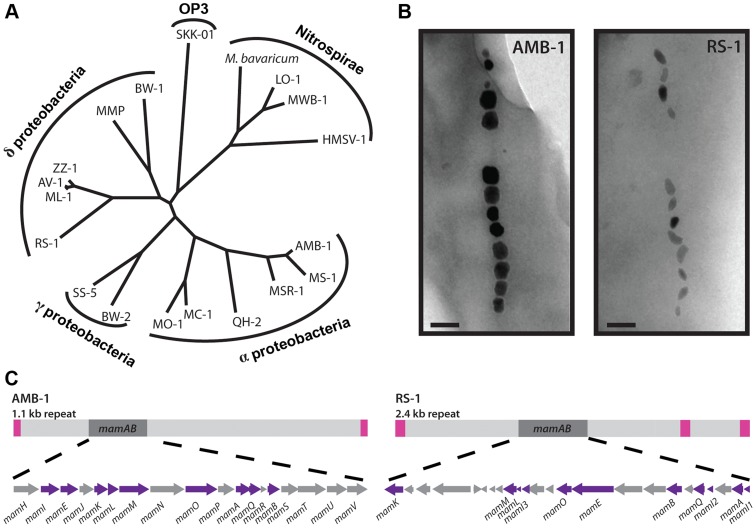
RS-1 is a representative of a group of bacteria that are phylogenetically and phenotypically distinct from the magnetotactic α-proteobacteria. A) 16S phylogenetic tree of magnetotactic bacteria. B) AMB-1 and RS-1 magnetite crystals visualized by TEM. Scale bar 100 nm. C) The *mamAB* gene clusters of AMB-1 and RS-1 shown in the context of the MAI. Pink squares represent the repeats surrounding each island. Purple arrows represent the genes that are conserved between the two.

Magnetotactic bacteria have been isolated all over the world, and belong to the α-, γ-, and δ-proteobacteria, as well as the Nitrospirae and candidate OP3 division (Figure 1A). Different species produce different types of magnetosomes, varying in their number, shape and size of crystal, organization within the cell, and mineral composition [8], [9]. In addition to the phenotypic diversity of the magnetosomes themselves, the magnetotactic bacteria are morphologically and physiologically diverse, with anaerobic and microaerophilic species; coccoids, vibrios, spirilla, and rod-shaped cells; and some obligate multicellular bacteria. Bioinformatic analyses of magnetotactic bacterial genomes have identified a genomic island that contains the genes for constructing magnetosomes [10], [11], termed the Magnetosome Island (MAI). While the MAIs of diverse magnetotactic bacteria vary in content, they share core magnetosome forming (*mam*) genes [11]–[13]. Though the magnetotactic bacteria are dispersed among many non-magnetotactic members of the Proteobacteria and related phyla, the *mam* genes suggest a monophyletic origin for the magnetosome trait. The phylogenetic trees of individual *mam* genes are the same as the phylogenetic trees of the ribosomal 16S sequences for the bacteria that possess them [14], suggesting either that the common ancestor of all Proteobacteria, Nitrospirae and the candidate OP3 division was a magnetotactic bacterium, or that the MAI was passed to some members of these groups via ancient horizontal gene transfer.

Mechanistic analyses of magnetosome formation have been conducted largely in two closely related model organisms, *Magnetospirillum magneticum* AMB-1 (AMB-1), and *Magnetospirillum gryphiswaldense* MSR-1 (MSR-1). Through biochemical [15]–[17] and genetic [18], [19] analyses, the *mam* and *mms* genes were identified as participating in magnetosome formation. These genes are located within the MAI, but they make up only a small portion of it. For example, the AMB-1 MAI is 98 kilobases (kb) long, but an 18 kb portion containing only the *mam* genes is able to partially substitute for the whole island [20], and 26 kb of the 115 kb MSR-1 island can reconstitute magnetosome synthesis in a non-magnetotactic bacterium [21]. Because much of the MAI encodes genes that are not required for magnetosome synthesis, it is not obvious which genes in an MAI from any given magnetotactic bacterium are participating in creating magnetosomes with different phenotypes.

AMB-1 and MSR-1 are α-proteobacteria, which produce cubo-octohedral shaped magnetite crystals, while the δ-proteobacteria, represented by RS-1, produce elongated bullet or tooth shaped crystals (Figure 1B). The MAIs of δ-proteobacteria have homologs to some of the α-proteobacterial MAI genes, but not others. Conspicuously absent are genes whose mutants have small and misshaped crystals, such as the *mms* genes and *mamS* (amb0975) [12]. For comparison, the AMB-1 and RS-1 islands are shown in Figure 1C. In addition to missing some α-proteobacterial genes, RS-1 contains genes that are shared exclusively among the MAIs of magnetotactic δ-proteobacteria, designated *mad* genes [11].

Microscopy studies have shown that AMB-1 and MSR-1 build membrane vesicles, called magnetosome membranes, which are then the site for magnetite synthesis [15], [18]. RS-1 contains numerous intracellular membranes [22], which makes looking for magnetosome membranes difficult. After release from iron starvation, RS-1 constructs another organelle composed of membrane-bound iron and phosphorus inclusions. In the same imaging study membranes were observed around these particles but not around magnetosomes [22]. These findings suggest that RS-1 may have a fundamentally different mechanism for constructing and maintaining its magnetic organelle.

These differences make a compelling argument for genetic and molecular analyses of magnetotactic δ-proteobacteria. Understanding the biological control of magnetite crystal shape and size is of particular interest to those designing magnetic nano-tools for industry [23] or health care [24]. The comparison of different systems that perform similar tasks has proved fruitful in other cases. For example research on different bacterial CRISPR systems resulted in the discovery of the simplified type II CRISPR that has become a valuable tool for manipulating eukaryotic genomes [25]–[27].

In this study we overcome the need for sophisticated genetic tools in RS-1 by taking advantage of modern sequencing methods to identify mutations from a classic forward genetic screen. We identified non-magnetic phenotypes for mutants of homologs of *mam* and *mad* genes, as well as genes not previously identified as being involved with magnetosome synthesis. These findings expand our understanding of magnetosome formation, both in the δ-proteobacteria and across all magnetotactic bacteria. Additionally, our results highlight the broad utility of this approach for studying interesting organisms that have previously been intractable to genetic analysis.

## Results

### RS-1 can be transconjugated with low efficiency

We chose RS-1 as a representative of the magnetotactic δ-proteobacteria because it can be grown as colonies on solid medium and in pure culture in liquid medium under conditions where magnetosomes are produced [22]. It also has a sequenced genome [12], consisting of a 5.2 megabase chromosome and two plasmids. In order to conduct genetic experiments, we attempted to transform RS-1 with a plasmid, pBMK7, that is designed for *Desulfovibrio* species [28]. We were successfully able to obtain RS-1 cells carrying pBMK7 by conjugation with *Escherichia coli*. However, the transconjugation rate was extremely low. With pBMK7, we obtained 10^−7^ transconjugates per recipient cell. For comparison, conjugation of AMB-1, an excellent genetic model system, yields 10^−3^ transconjugates per recipient cell [19], which results in thousands of transposon insertions per conjugation and allows for efficient screening of the entire genome.

The most comprehensive genetic analyses of the α-proteobacteria have come from genetic dissections of the AMB-1 and MSR-1 MAIs [29]–[31]. Systematic deletions have identified minimal sets of genes required to make magnetosomes, as well as assigned genes into groups that perform the functions of membrane remodeling, crystal nucleation, and crystal maturation. However, the tools available to create targeted deletions rely on suicide vectors that cannot replicate in the target organism such that DNA uptake and integration on the chromosome, two rare events, are selected for in one step. Attempts to move a suicide vector into RS-1 were unsuccessful, due to the low transconjugation efficiency and perhaps also a low frequency of homologous recombination.

An alternative is a genetic screen where genes are disrupted randomly and mutants with interesting phenotypes are selected from a large pool. However, modern bacterial genetic screens are performed with transposon-based tools that allow for easy identification of the disrupted gene but are delivered on suicide vectors. Attempts to obtain mutants with a range of mariner, Tn5, Tn7, and Tn10 based transposons were also unsuccessful. To work around this inability to generate transposon insertions, we isolated non-magnetic mutants created by chemical and UV mutagenesis, then used whole genome sequencing to identify the causative genetic change.

### Isolation of non-magnetic mutants

RS-1 has a doubling time of 11 hours and is an obligate anaerobe, though in our hands it is somewhat aerotolerant, and there is some evidence that it can survive near the oxic-anoxic transition zone in a gradient [32]. Screening large numbers of colonies is impractical, so we employed a two-step process. First a selection in liquid increased the proportion of non-magnetic cells in the population, then single colonies were screened for non-magnetic phenotypes. This strategy is similar to one previously used with AMB-1 [18]. To ensure that mutants with magnetosome-synthesis defects would not be accidentally discarded as magnetic because they still possessed magnetic crystals synthesized prior to mutagenesis, we began with non-magnetic cells, obtained by passaging wild type (WT) cells without iron.

We next wanted to verify the loss of magnetic particles in our cells due to this growth condition. The ability of RS-1 cells to synthesize magnetosomes can be measured by quantifying cellular alignment with a magnetic field rotated 90 degrees, using a spectrophotometer. The ratio of optical densities is called the coefficient of magnetism, or C_mag_ [33]. WT RS-1 has a C_mag_ between 1.4 and 1.6. After two passages without iron we found that the culture had a C_mag_ of 1, indicating that the cells no longer responded to a magnetic field. These WT, non-magnetic cells were then mutagenized with either ultraviolet radiation or ethyl methanesulfonate. In order to ensure that a sufficient level of mutagenesis was occurring, each mutagen was provided at a dose that resulted in 50% cell survival. As described in Table 1, this dose resulted in some mutants with only one genetic change, and others with dozens of changes when compared to the WT.

**Table 1:** The mutations and phenotypes of single-gene mutants.

Gene Number	Gene Name	Allele	Nucleotide change	Amino acid change	Cmag	Electron Dense particles by TEM	Other changes in genome
	WT RS-1				+++	Many bullet-shaped particles	
DMR_40800	*kup*	1	4619085 removed	Frameshift at position 217	+	Rare particles	0
		2	C4618459T	Premature stop at position 425	+	Rare particles	15
		3	G4618844T	Q297K	+	Rare particles	2
DMR_41030	*mamL*	1	C4639766T	E63K	+	Rare particles	3
		2	T4639844A	I37F	+	None observed	5
		3	C4639804T	G50E	+	None observed	ND
DMR_41050	*mad6*	1	G4640709A	Premature stop at position 177	++	Rare particles	1
DMR_41090	*fmpA*	1	A4645685T	D369E	++	Rare particles	3
		2	G4645540A	Premature stop at position 418	−	Rare round-shaped particles	1
DMR_41100	*fmpB*	1	G inserted after 4647284	Frameshift at position 270	++	Rare particles	1
		2	4647285 removed	Frameshift at position 270	++	Rare particles	2
DMR_41110	*mamB*	1	transposon	Transposon insertion	−	None observed	0
		2	C4648956T	C9Y	−	None observed	2
		3	C4648523T	Premature stop at position 153	−	Non observed	1
		4	C4648923T	G20D	++	Rare particles	20
		5	C4648888T	G32S	−	None observed	8
DMR_41120	*mad2*	1	C4649529T	Premature stop at position 37	−	Rare jagged particles	12
		2	C4649155T	G162S	−	Rare round-shaped particles	1
		3	G4649338A	H101Y	−	Rare round or jagged particles	2
DMR_41130	*mamQ*	1	G4650091A	Premature stop at position 65	−	None observed	23
		2	G4649752A	P178S	−	None observed	6
		3	C4649805T	R160Q	−	None observed	1
DMR_41150	*mad1*	1	G4650957A	R283W	−	Rare jagged particles	1
DMR_41280	*tauE*	1	G4664110T	Premature stop at position 226	−	None observed	0
		2	C4664713T	G26D	−	None observed	8
		3	transposon	Transposon insertion	−	None observed	2

+++ indicates a C_mag_ of 1.4–1.6, ++ a C_mag_ of 1.1–1.3, + that some cultures have a C_mag_ around 1.05, near the limit of detection, and – a C_mag_ of 1.

ND: *mamL* allele 3 was the only mutant not analyzed by whole genome sequencing; its mutation was discovered by PCR and Sanger sequencing.

**Figure 2:**
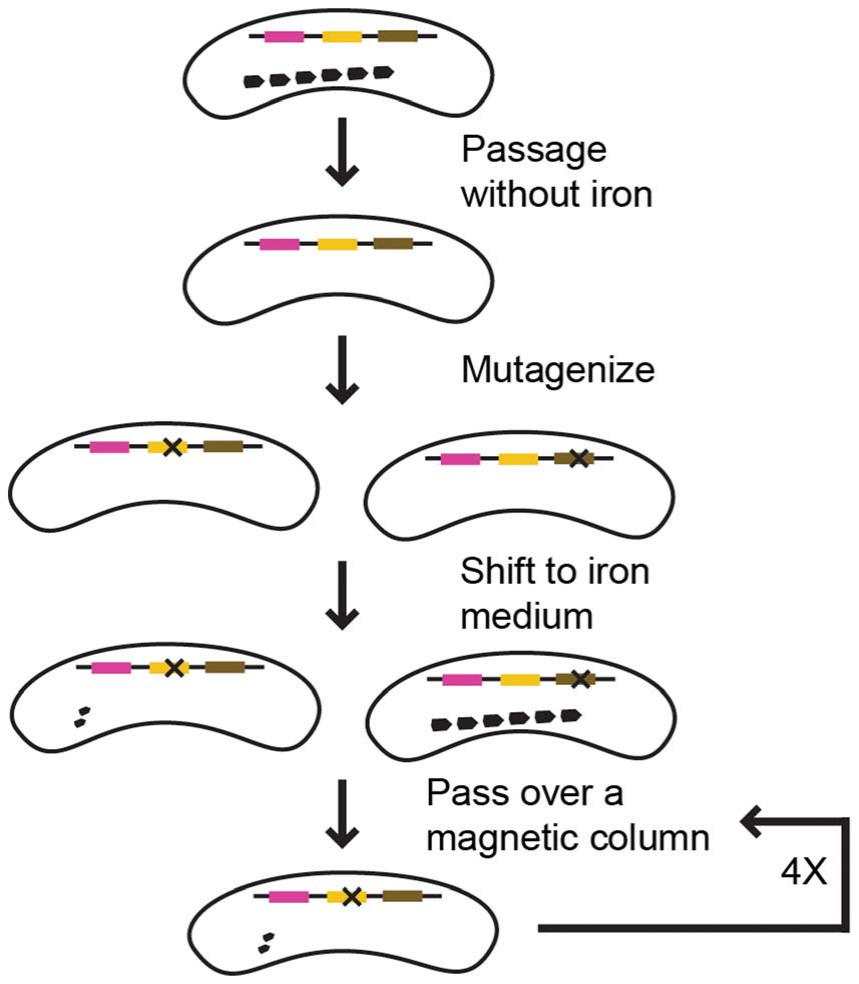
Screen strategy. Pink, yellow, and brown lines represent genes. “X” represents a mutation. In this example, a mutation in the yellow gene resulted in a magnetic defect, whereas a mutation in the brown gene did not.

After mutagenesis, the cells were inoculated into fresh growth medium with iron. We expected the resultant culture to contain a mixture of mutant cells, most of which would be magnetic. To isolate the rare non-magnetic mutants, we passed the culture over a magnetized column and collected the flow-through, which contained non-magnetic cells. However, much of the flow-through was only transiently non-magnetic, and when inoculated into fresh media with iron resulted in a culture with a WT C_mag_. This suggests that a proportion of a WT RS-1 culture is either naturally non-magnetic, or lags in magnetosome formation after release from iron starvation. To isolate those cells that had a true non-magnetic phenotype, we repeated the outgrowth and column selection several times. With each repetition, the C_mag_ of the outgrowth decreased, indicating non-magnetic mutants were taking over the population. After four rounds of growth and selection the column flow-through was plated on solid media and single colonies were picked for further analysis. Those colonies with non-magnetic or low-magnetic phenotypes were analyzed by whole genome sequencing to determine the causative genetic change. The screen strategy is described in Figure 2.

Because of the number of outgrowths, we were concerned that identical mutant colonies could be isolated that all descended from one mutagenized cell. To avoid this, the outgrowth process was performed on many independent cultures, and only one colony from each culture was analyzed. After the mutation for each strain was identified, we used PCR and Sanger sequencing to check for this change in the other strains isolated from the same outgrowth. We then analyzed those strains that were not clones by whole genome sequencing to determine their mutation. One strain contained a different mutation in the same gene, and was included in this study without having its whole genome sequenced (*mamL* (DMR_41030) allele 3).

In addition to deletions of the entire MAI, we characterized 29 mutants, which are summarized in Tables 1 and S1. They include three large deletions and mutations in ten single genes. Two mutations were caused by the insertion of a transposon into coding sequences. This transposon, consisting of a gene with similarity to the IS5 family transposases, exists naturally in RS-1, and appears to have mobilized during the mutagenesis and selection process. There are 13 copies of this gene in the published genome sequence of RS-1, including one located in the MAI region between groups I and II (*DMR_41190*).

The mutants had a variety of magnetic phenotypes, categorized into three groups: (1) a completely non-magnetic phenotype, (2) a very limited magnetic response with some replicate cultures displaying a C_mag_ near the limit of detection and others appearing to have no magnetic response, (3) a consistently measurable C_mag_ below that of WT. These phenotypes are described as −, +, and ++, respectively, in Table 1. Three genes have been named in this study. *DMR_41280* has been named *tauE*, based on its gene product's membership in the Domain of Unknown Function (DUF) 81 TauE protein family. *DMR_41090* and *DMR_41100* have previously been assigned names, but for reasons outlined in the discussion are renamed here *fmpA* and *fmpB*, respectively, for Fewer Magnetic Particles.

### Magnetosome genes form a genomic island that can be lost in RS-1

**Figure 3:**
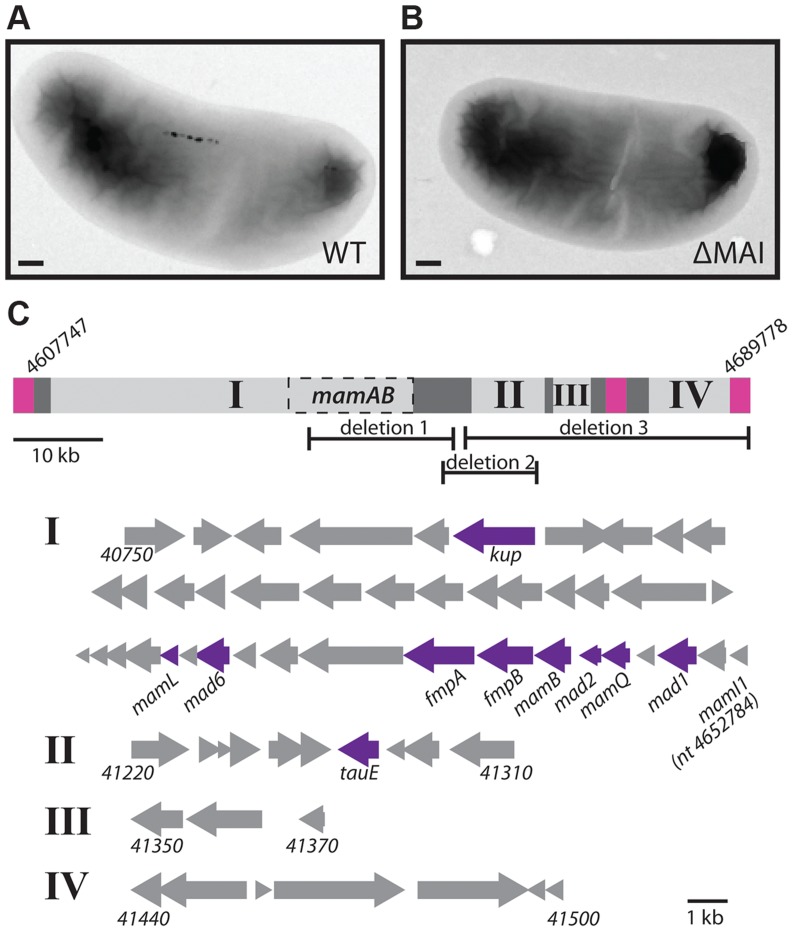
The RS-1 MAI. TEM of WT (A) and MAI deletion (B) cells. Scale bar 200 nm. C) Depiction of groups I, II, III, and IV of the MAI. Pink squares represent the repeats surrounding the island. Dark grey represents areas of transposons and repeats. Dashed box labeled MamAB is the region detailed in figure 1C. The nucleotide positions of the beginning and end of the island are indicated. The areas of the island missing in deletions 1, 2, and 3 are also shown. Below, the genes that make up groups I, II, III, and IV are represented by arrows, with the beginning and ending gene names indicated for each group. The ending nucleotide position is given in the case of *mamI1*, which is not assigned a gene number. Genes that were hit in the screen are indicated in purple.

Many magnetotactic bacteria organize magnetosome synthesis genes into a genomic island, the MAI. The MAI is frequently surrounded by repetitive sequence elements that enable its excision and loss from the chromosome by homologous recombination [10], [34]. In the laboratory model systems AMB-1 and MSR-1 spontaneous island deletions are often isolated during normal laboratory cultivation. As with other magnetotactic bacteria, the genome sequence of RS-1 reveals a cluster of magnetosome-related genes surrounded by repetitive sequence elements. The RS-1 MAI is an 82 kb region of DNA between nucleotides 4607747 and 4689778 [12], [17]. Flanking this region is a 2.4 kb direct repeat (Figure 3C, pink regions) that consists of three genes with homology to the IS66 family of insertion sequence elements. There are a total of 12 of these IS66 operons located on the RS-1 chromosome and plasmids, including two other pairs that have 100% nucleotide identity to the pair that flanks the RS-1 MAI.

Approximately 90% of mutants recovered in this work were island deletions. Island deletions had no coverage at positions within the MAI when analyzed by whole genome sequencing (Figure S1). The deletion of the MAI was confirmed by the ability to obtain PCR products using primers just outside, but not just inside the border of the MAI. RS-1 MAI deletions could be isolated by the non-magnetic enrichment and outgrowth method used in this screen even in the absence of mutagenesis, suggesting that island loss is occurring naturally in RS-1. RS-1 MAI deletions lack the electron-dense particles found in the WT (Figure 3A and B) and have no magnetic response (C_mag_ = 1).

In addition to the 2.4 kb repeat, portions of the MAI consist of DNA transposases, recombinases and short repetitive sequences (Figure 3C, dark grey regions). These areas divide the remaining genes, which may function in magnetosome synthesis, into four groups, indicated in Figure 3C by Roman numerals. The largest of these, group I, contains most of the genes that have homology to genes in other magnetotactic bacteria. These include all the *mam* genes, which are common to magnetotactic bacteria [11]–[13], and some of the *mad* genes, which are shared among the magnetic δ-proteobacteria [11].

Groups II and III contain some *mad* genes (*mad5 (DMR_41230*) and *mad12* (*DMR_41300)*), as well as some genes that share homology with signaling or transcriptional regulation genes (*DMR_41220*, *DMR_41310*). Group IV contains genes associated with motility, such as a *cheW* homolog and genes encoding a methyl accepting chemotaxis protein and a GGDEF domain protein (*DMR_41440*, *DMR_41450*, and *DMR_41480*, respectively). There is a copy of the 2.4 kb repeat that has 94% nucleotide identity to those surrounding the MAI and is located directly upstream of group IV, suggesting that these genes might compose a motility module that could be lost by recombination, though this was not observed during this study.

Three mutants with deletions of a portion of the island were recovered (Figure 3C, Table S1). In each case the region between groups I and II formed one edge of the deletion. This region contains many 46 base, 12 base, and 10 base direct repeats; however these repeats do not border the deletions, suggesting they were not created by homologous recombination with the repeats. In deletion 1, a portion of group I is missing, removing many *mam* genes, such as *mamB* (*DMR_41110*) and *mamQ* (*DMR_41130*), whose deletions in the α-proteobacteria result in non-magnetic cells [29], [30]. The other two deletions remove areas outside of group I. One includes most of group II, and the other includes the entire right side of the island, groups II, III, and IV. All three deletions have non-magnetic phenotypes, with a C_mag_ of 1 and no particles visible by transmission electron microscopy (TEM, Figure S2). These results show that there are genes in addition to the *mam* and *mad* genes of group I that are necessary for RS-1 to form magnetite crystals.

### RS-1 intracellular membranes and iron-phosphorus organelles do not require the MAI

RS-1 cells are filled with intracellular membranes [22]. Intracellular membranes are rare among bacteria, often being involved in photosynthesis or energy production [35]. As RS-1 does not photosynthesize, the function of its extensive intracellular membrane network remains mysterious. In our previous work we were not able to see membranes surrounding RS-1 magnetite crystals, even though many intracellular membranes are visible throughout the cell and surrounding the iron-phosphorus inclusions that RS-1 transiently produces upon release from iron starvation [22]. However, it is possible that membranes do surround RS-1 crystals, but that they are so closely associated that they are not visible by microscopy. Indeed, Matsunaga and coworkers observed some material by TEM around purified magnetosomes that was removed by detergent treatment, which they interpreted as magnetosome membranes [17]. Some of the intracellular membranes of RS-1 have an elongated shape that vaguely resembles the elongated shape of RS-1 magnetite crystals although their dimensions are significantly different. In previous work no magnetite crystals were observed within these intracellular membranes yet a connection between them and magnetite formation could not be ruled out [22].

**Figure 4:**
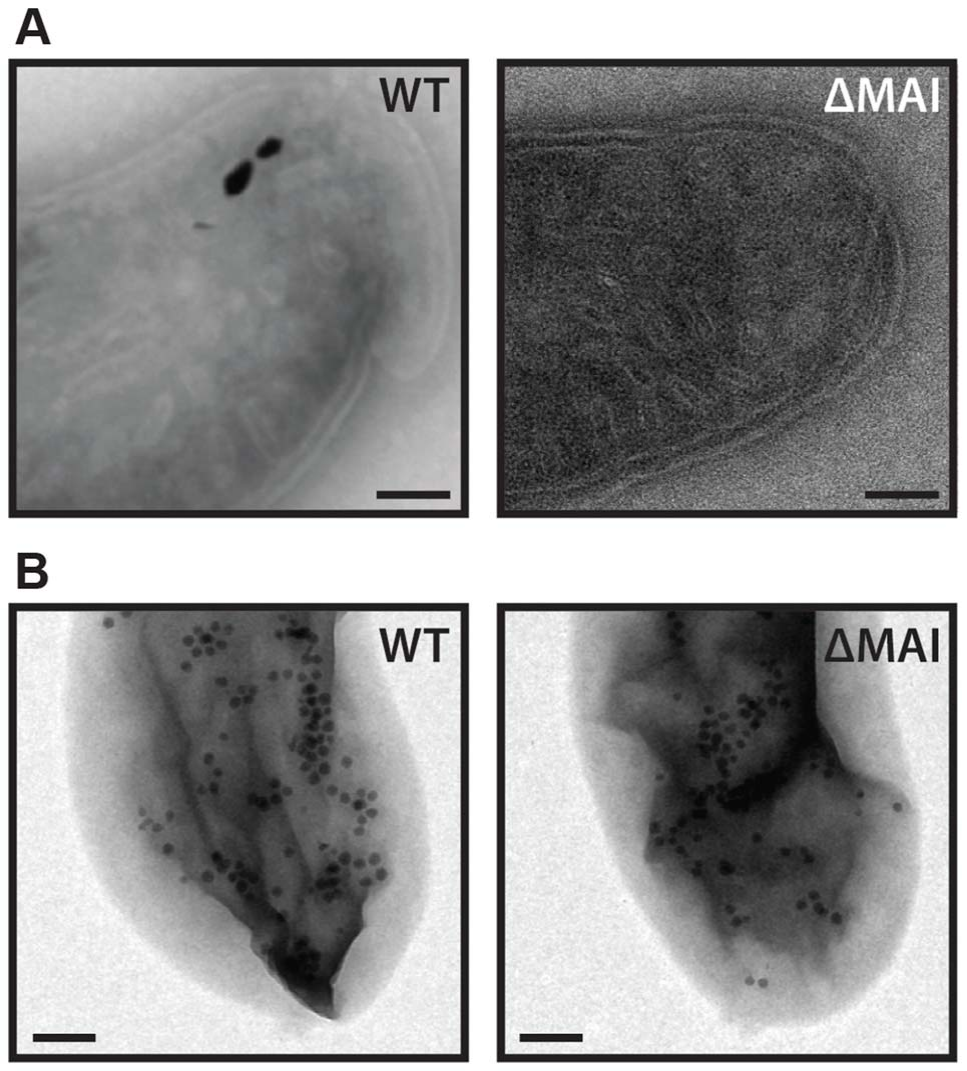
RS-1 MAI deletion cells still make intracellular membranes and iron-phosphorus organelles. A) Intracellular membranes in WT and MAI deletion RS-1. Scale bar 100 nm. B) iron-phosphorus organelle production was induced by the reintroduction of iron to iron-starved cells. Samples imaged 3 hours after induction. Scale bar 200 nm.

In the α-proteobacteria, the genes for remodeling the inner membrane to produce magnetosome membranes are encoded within the MAI, and in the absence of these specific genes or of the entire MAI no magnetosome membranes are synthesized [29]. To determine if the intracellular membranes of RS-1 depend on the MAI, we compared an MAI deletion strain to WT by preparing the cells using cryo-ultramicrotomy and imaging them with TEM. As shown in Figure 4A, both strains contain elongated intracellular membrane structures, suggesting that RS-1 magnetosomes and intracellular membranes are unrelated. It is possible, however, that RS-1 synthesizes crystals using membranes that were native to the bacterium before the acquisition of the MAI, or that the genes responsible for creating and maintaining the intracellular membranes were originally part of the MAI but have since been moved to a different part of the genome.

In addition to making magnetosomes, RS-1 makes an iron and phosphorus containing organelle [22]. In the hours after release from iron starvation, hundreds of intracellular nanometer-sized iron phosphorus particles surrounded by membranes appear throughout the cell, and then disappear over the course of three days [22]. As their disappearance corresponds with the synthesis of magnetosomes, we initially hypothesized that they were magnetosome precursors. However, a pulse-chase experiment showed that the iron in the iron-phosphorus organelles did not end up in magnetosome crystals [22], suggesting that they are unrelated to magnetosome synthesis. To determine if the iron-phosphorus organelles require magnetosome genes, we subjected WT and MAI deletion cells to iron starvation, and then followed them by TEM after the re-introduction of iron. As shown in Figure 4B, both strains produced the iron-phosphorus organelles, confirming our original finding that these are unrelated processes that occur in RS-1.

### The mutants are complemented with WT MAI genes

**Figure 5:**
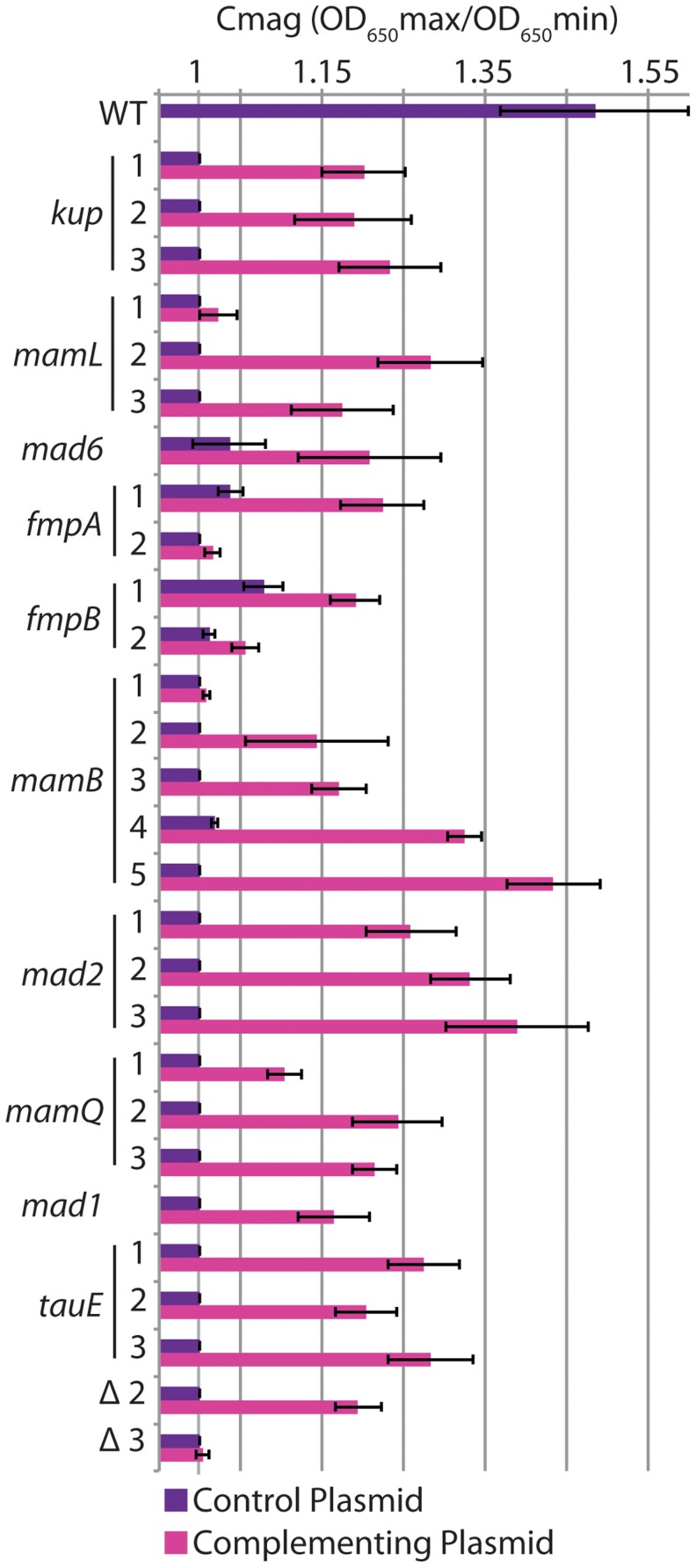
Complementation of mutants. Strains were transformed with an empty plasmid control (purple bars) or a plasmid expressing the gene mutated in each strain (pink bars). Error bars indicate the standard deviation from multiple replicates (for details see table S3).

Some of the isolated mutants differed from the WT genome sequence at only one position, while others contained many changes (Tables 1, S2). In these cases, we predicted that the change in a gene located in the MAI was responsible for the observed phenotype. To test this, we complemented each of the mutants by expressing a WT copy of the mutated MAI gene from plasmid pBMK7. Some genes were expressed from the RS-1 *mamA* (*DMR_41160*) promoter, while others were expressed from their own promoters (Tables S3, S4). Though complementation efficiency differed, expression of the candidate MAI gene increased the C_mag_ in all cases compared to the same mutant that was carrying the empty vector (Figure 5).

In some cases different mutant alleles of the same gene exhibited varying levels of complementation. For instance, unlike other alleles of *mamB*, allele 1 was complemented at a very low level. This allele is a transposon insertion that may have polar effects on downstream genes. By contrast, the transposon insertion in *tauE* allele 3 complemented to the same level as the other *tauE* alleles. The gene downstream of *tauE* is on the opposite strand, so in this case no polar effects would be predicted. *mamL* allele 1 (E63K), and *fmpB* allele 2 (a frameshift) could not be complemented even though the other alleles of *mamL* and *fmpB* could, suggesting that these are dominant negative alleles. Alternatively, one or a combination of the three or two additional mutations in these respective strains could be responsible for the phenotype. Surprisingly, *fmpB* allele 2 contains a frameshift at the same amino acid as *fmpB* allele 1, which is complementable. The alleles are the gain (allele1) or loss (allele 2) of an extra cytosine in a poly-cytosine tract, and encode proteins that end with different polypeptides. These may impart different biochemical properties on the FmpB mutant proteins, perhaps resulting in degradation of one and toxic build up of the other.

*tauE* was the only single gene we found outside of group I whose mutants have a non-magnetic phenotype. The location of *tauE* could account for the phenotypes of large deletions 2 and 3, both of which lack *tauE*. We investigated whether *tauE* was sufficient to complement the loss of most of group II or the loss of groups II–IV. We found that *tauE* alone was able to complement deletion 2 to a similar level as single *tauE* mutations but had little effect on the magnetic response of deletion 3 (Figure 5). This indicates that while much of group II besides *tauE* is dispensable for magnetosome formation, there is another factor or factors in groups III and IV that are required for magnetosome synthesis in RS-1 but were not identified in this screen.

### Point mutations in *mamB* and *mamL* highlight functional regions of the encoded proteins

**Figure 6:**
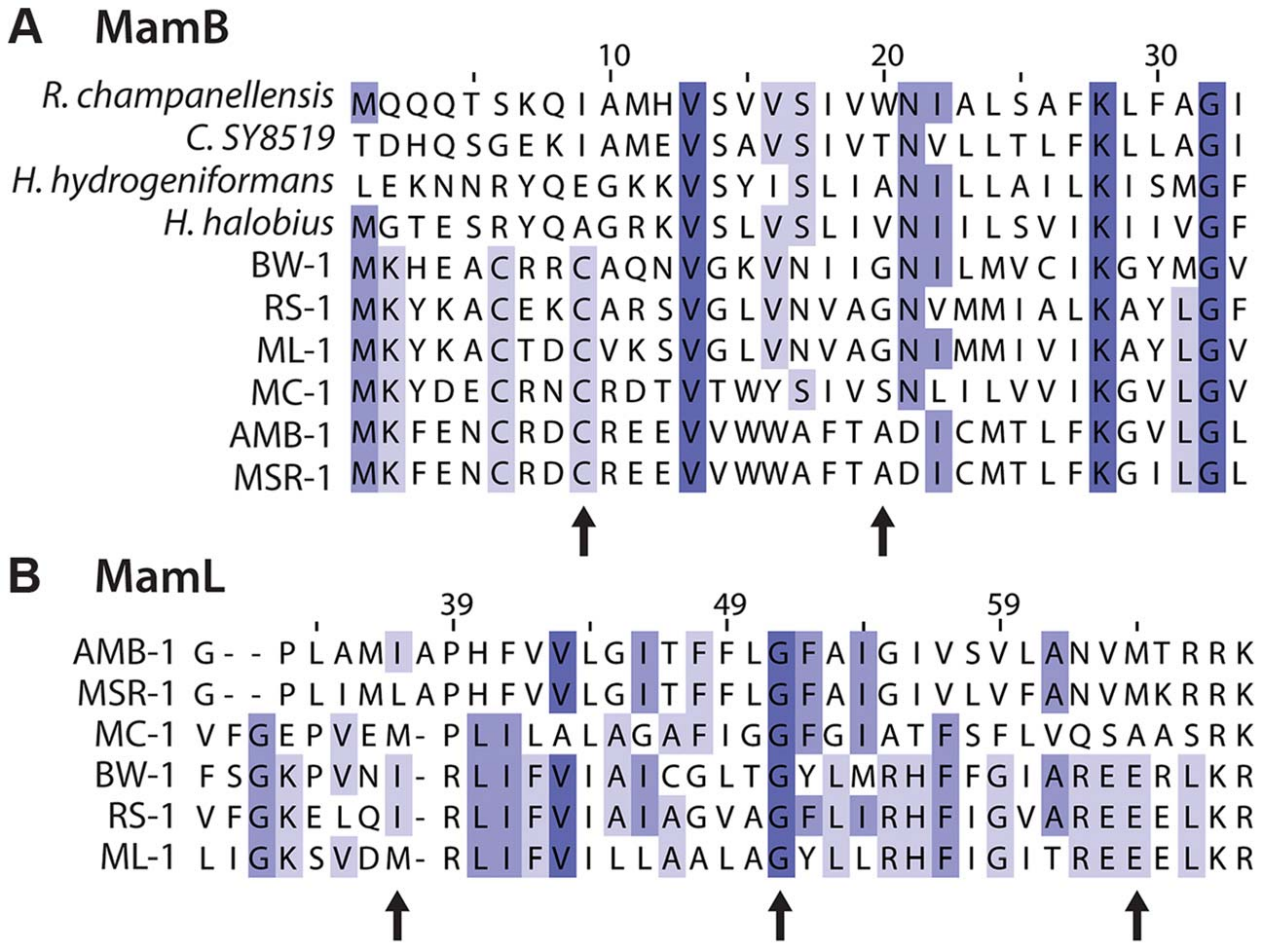
Details of multiple sequence alignments of magnetosome proteins. BW-1, RS-1 and ML-1 are δ-proteobacteria. AMB-1, MSR-1 and MC-1 are α-proteobacteria. Purple shading indicates percent conservation among the aligned sequences. Arrows indicate amino acids discussed in text. A) Alignment of MamB. Several of the most closely related CDF domain proteins from non-magnetotactic bacteria are also included: *Ruminococcus champanellensis* (E value 1×10^−43^, NCBI accession CBL18105), *Clostridium SY8519* (E value 1×10^−42^, NCBI accession BAK46352), *Halanaerobium hydrogeniformans* (E value 3×10^−44^, NCBI accession ADQ15651), and *Halobacteroides halobius* (E value 1×10^−43^, NCBI accession AGB41459). B) Alignment of MamL, which only has homologs among magnetotactic bacteria. The position number of the alignment is not the same as the residue number for RS-1 MamL.

Due to their functional importance in the α-proteobacteria and their conservation in every magnetotactic bacterium investigated, we expected to find some *mam* gene mutants with non-magnetic phenotypes. We isolated mutations in *mamB*, *mamL*, and *mamQ*. MamB is a member of the cation diffusion facilitator (CDF) family, which exports divalent cations from cells. In magnetosome synthesis MamB and another CDF protein, MamM, are thought to be involved in the transport of iron [36]. MamB is also required for magnetosme membrane formation in AMB-1 [29]. Five alleles of *mamB* were isolated, four with non-magnetic phenotypes. As shown in Figure 6A, G20 is conserved among the δ-proteobacterial MamBs. MamB^G20D^ has a less severe phenotype than the other alleles of *mamB* suggesting that this residue may not be required for MamB function, even among the δ-proteobacteria. MamB^C9Y^ is of particular interest as the mutated cysteine, along with neighboring residues C6 and K2, is conserved across all magnetosome MamB proteins, but varies among CDF family homologs from other species (Figure 6A). Together with the non-magnetic phenotype of MamB^C9Y^, this conservation pattern suggests the amino-terminus of MamB is important to its role in magnetosome formation.

We isolated three *mamL* alleles, which initially appeared to have no magnetic response, as shown in Figure 5. However, occasional cultures of all three *mamL* mutants had a C_mag_ of about 1.05, nearing our limit of detection. We interpret this to mean that these mutants are so impaired in magnetosome formation that only rare cultures had enough magnetic cells to produce a measureable C_mag_. This is supported by the rare particles observed in some cells of *mamL* allele 1 by TEM (Figure S2). Though no particles were observed for the other alleles of *mamL*, these strains' occasional magnetic response implies that crystals can be present, though not observed in our experiments. In the α-proteobacteria, *mamL* deletion results in complete loss of crystals and magnetosome membranes [29]. Together, this suggests that either all three alleles isolated for *mamL* retain partial function, or *mamL* is not required for the synthesis of magnetite in RS-1.

The *mamL* alleles are all point mutations. As shown in Figure 6B, the I37 position contains mainly aliphatic amino acids, but in mamL^I37F^ an aromatic phenylalanine is at this position. RS-1 E63 is in a group of acidic amino acids that are present only in δ-proteobacterial MamLs, but in mamL^E63K^ this acidic stretch is disrupted with a basic lysine residue. The conservation pattern of this acidic stretch and its phenotype in RS-1 points to this area of the protein as being important for MamL's ability perform δ-proteobacteria-specific functions.

### Some *mad* genes have non-magnetic phenotypes

The *mad* genes were recently identified using comparative genomics. They are defined as being conserved uniquely in the MAI regions of magnetotactic δ-proteobacteria [11]. It was hypothesized that due to this conservation pattern they must be important for magnetosome formation in the δ-proteobacteria. However, no *mad* gene phenotype has been observed and nothing is known about their activity or function. 31 *mad* genes were identified, and RS-1 has homologs of 25 of them [6], [11]. We were interested in whether the screen uncovered mutations in any *mad* genes that resulted in non-magnetic phenotypes. Indeed, mutants of *mad1* (*DMR_41150*), *mad2* (*DMR_41120*), and *mad6* (*DMR_41050*) were isolated. This is the first demonstration of phenotypes for *mad* genes.

The *mad6* mutant had a limited magnetic response. Mad6 consists mostly of a NapH nitrate reductase domain, though RS-1 does not reduce nitrate [37]. In MSR-1, deletions of genes affecting nitrate reduction have effects on magnetite biomineralization [38], [39]. Mutations of *mad1* and *mad2* resulted in cells with no magnetic response. Mad1 and Mad2 have no obvious homology to any characterized proteins. Mad1 contains three CXXCH heme-binding motifs. Two adjacent CXXCH motifs is a feature common to a number of magnetosome proteins, and has been termed the magnetochrome domain [40], [41]. The sequences of Mad1 and Mad6 suggest they both perform redox functions, a feature found in a number of magnetosome proteins from the α-proteobacteria. This aspect of the δ-proteobacterial genes *mad1* and *mad6*, as well as *fmpB* (described in the discussion), suggest that redox activity is just as important to magnetosome synthesis, if not more so, in the δ-proteobacteria.

### Some mutants synthesize misshaped particles

**Figure 7:**
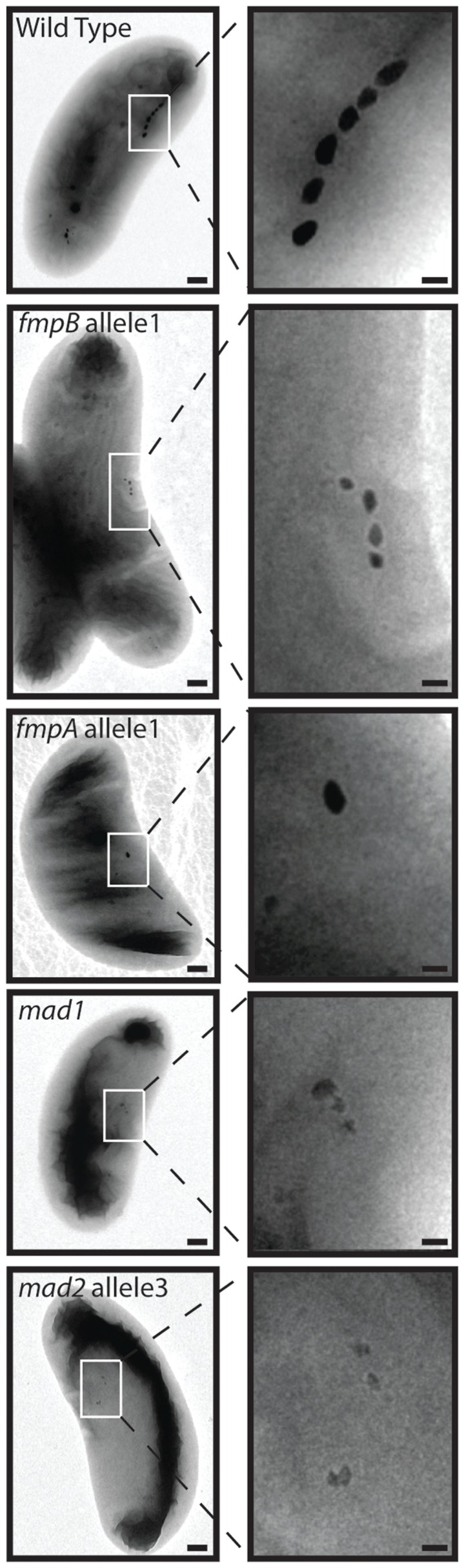
Electron-dense particles are found in some mutants. TEM of WT and mutant cells show WT-like particles in some mutants (*fmpB*, *fmpA*) and unusual-looking particles in others (*mad1*, *mad2*). Scale bar 200 nm for whole cell images, 50 nm for enlargements.

Given the unique morphology of RS-1 crystals, we were particularly interested in mutants that produced misshaped magnetic particles. Thus, we examined the mutants with whole-cell TEM. As described in Table 1 and shown in Figures 7 and S2, some non-magnetic mutants contained no electron-dense particles. This suggests that MamB, MamQ, and TauE are absolutely required for magnetite synthesis.

As expected, mutants with low C_mag_ values were found to contain electron-dense particles consistent with magnetite. As observed above for *mamL* allele 1, the shape, size, and organization of these particles appear similar to WT; however the number of particles per cell was much lower (Figures 7, S2). In mutant cultures with C_mag_ values above one, most cells had no particles. Those mutant cells that had particles contained between one and four per cell, while WT RS-1 cells have 10+/−4 crystals per cell. These results indicate that Kup, MamL, Mad6 and FmpB contribute to efficient magnetite formation, but that WT-like crystals can be produced at a lower rate if they are impaired.

In contrast, *mad1* and *mad2* mutants and cells with *fmpA* allele 2 contained rare, electron-dense particles even though they have no magnetic response. These particles appeared less elongated than in the WT (Figure 7 *mad1*), and they sometimes had sharp corners or jagged-looking edges (Figure 7 *mad2* allele 3).

### Mad1 is required for stable, single-domain magnetite crystallization

**Figure 8:**
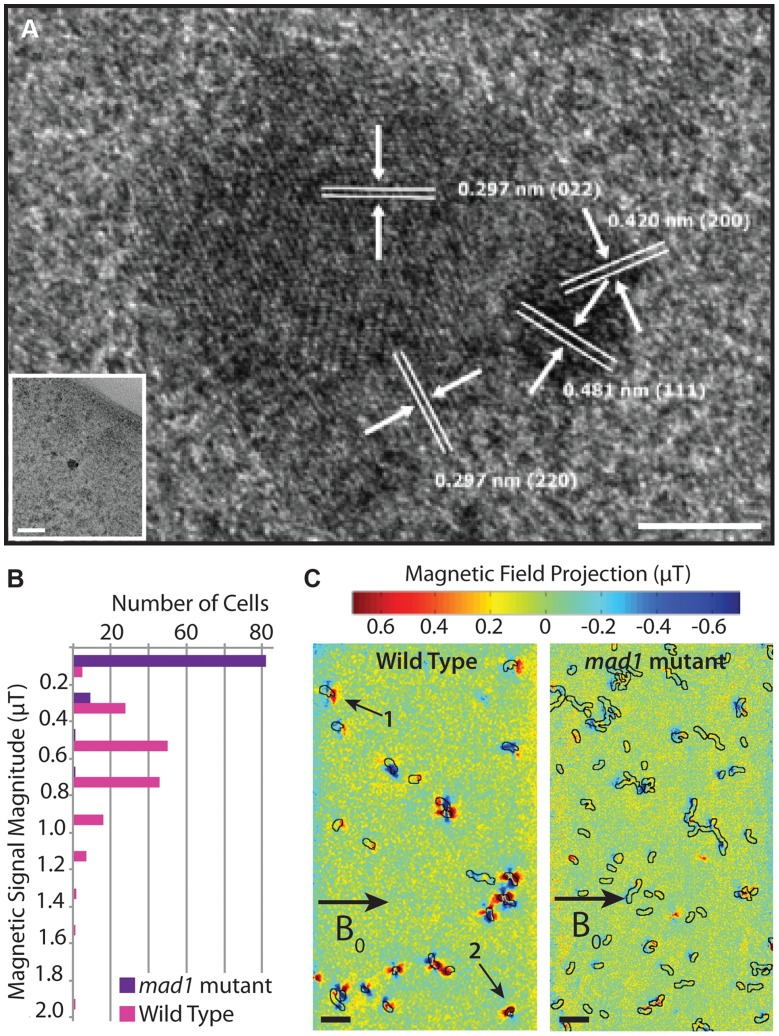
*mad1* mutants produce paramagnetic, multi-domain magnetite crystals. A) *mad1* mutant crystal. Lattice measurements indicate that the crystal is magnetite. Several crystal domains are present. Scale bar 5 nm, inset scale bar 50 nm. B) Distribution of magnetic field magnitudes recorded from WT and *mad1* mutant RS-1 cells using nitrogen-vacancy (NV) diamond-based magnetic imaging in a 1.2 mT external bias field. The WT cells produce magnetic fields on the order of 0.6 µT, whereas only a small fraction (∼10%) of the mutants produce magnetic fields distinguishable from zero in these measurements. C) Magnetic field images of WT (left panel) and *mad1* mutant (right panel) cells recorded with a 20.5 mT external bias field. The superimposed black outlines indicate the cell boundaries, as determined from optical transmission images. The fields shown here are one scalar component of the total vector magnetic field, projected along the direction of the bias field (indicated by the arrow labeled B_0_). The WT cells show field patterns consistent with randomly oriented permanent dipoles; cells labeled 1 and 2 are examples of dipoles with clearly distinguishable orientations, the latter at a significant angle to the external bias field. The mutant cells show weaker field patterns in general, and dipoles are preferentially aligned parallel to the direction of B_0_ (negative lobe on the left, positive lobe on the right), consistent with a paramagnetic response to the external field. Scale bars 10 µm.

We wondered why some mutants, such as the *mad1* mutant, have no magnetic response despite the presence of electron-dense particles. To detect if the particles in *mad1* mutants are magnetite, we performed high-resolution transmission electron microscopy (HRTEM) on these samples, finding small, multi-domain crystals (Figure 8A). The different angles of the lattice lines in Figure 8A indicate that the crystal is composed of multiple domains, and the crystal lattice line spacing indicates that the crystal is magnetite. This is reminiscent of the phenotype of a deletion of *mamS*, a gene found in AMB-1 but absent in RS-1, where many small crystals are found together in one magnetosome membrane [29]. These results suggest that Mad1 may regulate crystal nucleation, limiting each developing crystal to one nucleus, or that it functions to cull multi-nucleate crystals down to one as the crystals grow in size.

The multi-domain nature of the magnetite crystals in the *mad1* mutant offers an explanation for why these cells are unable to turn in a magnetic field. It is thought that the many small, aligned magnetic moments of the magnetite crystals in magnetotactic bacteria combine to produce a magnetic moment strong enough to turn the cells in a magnetic field [42]. As the crystals of *mad1* mutant cells are multi-domain, each domain may be too small to hold a stable dipole moment. Or, if large enough, the unaligned magnetic moments may work against each other. To observe the effect this has on the magnetic moment of the entire cell, we employed a wide-field optical magnetic microscope using nitrogen-vacancy (NV) color centers in diamond [43] to observe the magnetic field produced by WT and mutant cells. This technique relies on the fluorescence produced by NV centers in diamond that have been spectroscopically probed by microwaves in order to visualize local magnetic fields under ambient conditions. Because most *mad1* mutant cells lack any electron-dense particles, we enriched the mutant population for those containing magnetite by passing the culture over a magnetized column and imaging only the 1–2% of cells that were retained on the column.

We carried out the initial NV-diamond magnetic imaging of wild-type RS-1 and *mad1* mutant cells in a small externally-applied bias field of 1.2 mT. WT cells dried on the surface of the diamond chip were measured with the NV technique, and found to produce dipolar magnetic fields on the order of 0.6 µT (consistent with an average magnetic moment in the range of 10^−18^ to 10^−17^ A m^2^, and approximately an order of magnitude smaller than the average magnetic moment measured for wild-type AMB-1 [43]), whereas almost none of the *mad1* mutant cells produced fields above the measurement noise floor of ∼0.1 µT (Figure 8B). To test for possible paramagnetic behavior, the same WT and mutant cells (in the same fields of view) were then measured at a higher bias field of ∼20.5 mT. In this case, only a scalar magnetic field projection along a single diamond crystal axis was measured, instead of the full vector field recorded in the first set of experiments. At the larger bias field, most WT cells still produced magnetic field patterns consistent with randomly oriented, fixed dipoles in the plane of the diamond (Figure 8C). However, a significant fraction of the *mad1* mutant cells now also produced dipolar field patterns, predominantly aligned along the direction of the applied bias field (Figure 8C). Approximately one third of the mutant cells produced detectable fields in the 20.5 mT bias, compared to less than 10% of the same population at 1.2 mT bias. This paramagnetic behavior is consistent with the formation of many small, superparamagnetic magnetite domains in the *mad1* mutants, instead of the larger, blocked ferrimagnetic particle chains observed in the WT cells.

### The RS-1 MAI-encoded Kup is a potassium transporter

Our screen for non-magnetic mutants uncovered two genes in the RS-1 MAI that are homologous to common bacterial genes. Mutations in *tauE* and *kup* (*DMR_40800*) resulted in non-magnetic phenotypes even though RS-1 has additional copies of each of the effected genes outside the MAI. *tauE* encodes a putative anion transporter [44] found in many bacteria. RS-1 has an island copy (*DMR_41280*) and an extra-island paralog (*DMR_40120*) of *tauE*. TauE domains are found fused to the protease domains of MamE in RS-1 (*DMR_41080*) and MamO in AMB-1 (amb0969), though no stand-alone TauE protein has been identified as being important for magnetosome synthesis. Like *tauE*, *kup* is a common bacterial gene of which RS-1 contains an MAI-specific paralog (*DMR_40800*) and an extra-island paralog (*DMR_15830*). We isolated three alleles of *kup_DMR_40800_*, all of which are associated with rare cells containing electron-dense particles and few cultures having a measurable C_mag_. Kup proteins are ubiquitous potassium transporters that have been characterized in *E. coli* and plants [45]. Most bacteria possess one copy of *kup*, though some, like RS-1, have two.

**Figure 9:**
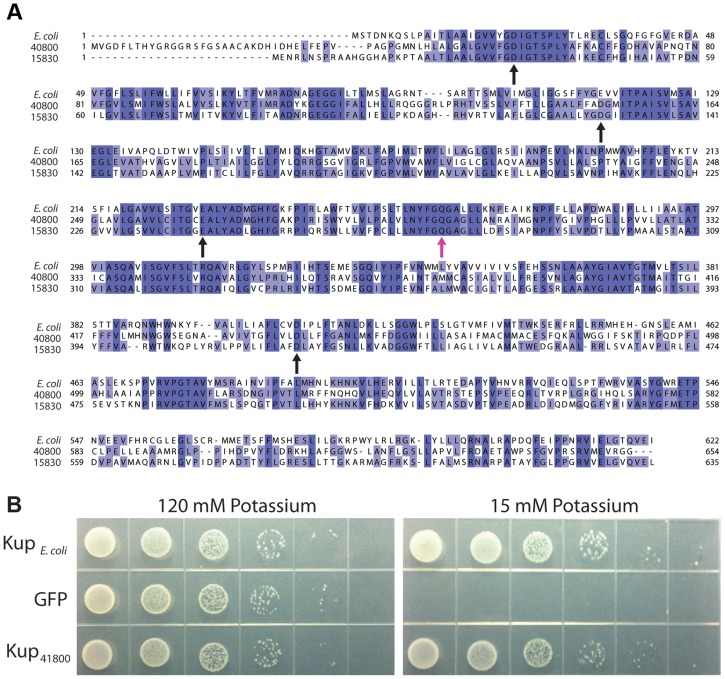
Kup_DMR_40800_ is a potassium transporter. A) Sequence alignment of the two RS-1 Kup proteins with Kup*_E. coli_*. Purple shading indicates percent conservation among the aligned sequences. Black arrows indicate the conserved residues that have been shown to be important for Kup function in *E. coli* [46]. Pink arrow indicates Kup_DMR_40800_ Q297, which is changed to K in allele 3. B) *E. coli* strain TK2446 expressing alleles of Kup or the control expressing GFP. Cells were grown in a potassium-rich media, washed to remove potassium, then plated on the indicated concentration of potassium as a 1∶10 dilution series.

We were surprised to find a phenotype for *kup_DMR_40800_*, as potassium transport has not previously been implicated in magnetosome synthesis. An alignment of RS-1 Kup proteins DMR_40800 and DMR_15830 with the well-studied Kup*_E.coli_* (YP_026244) shows a significant amount of conservation (Figure 9A). Though the structure and mechanism of potassium transport for Kup remain undetermined, a recent study highlighted several negatively charged amino acids that are required for transport in *E. coli*, D23, E116, E229, and D408 [46]. As shown in Figure 9A, these are also conserved in both RS-1 proteins, consistent with the idea that *kup_DMR_40800_* encodes a potassium transporter.

To see if Kup_DMR_40800_ can transport potassium, we tested whether it was able to rescue the growth of a strain of *E. coli*, TK2446, in which all potassium transporters have been deleted. TK2446 is capable of growth in the presence of high potassium (120 mM), but requires the expression of a potassium transporter to grow in the presence of low potassium (15 mM). As shown in Figure 9B, expression of Kup*_E. coli_* allows TK2446 to grow on low potassium, while cells carrying the control vector that expresses Green Fluorescent Protein cannot grow. As with Kup*_E.coli_*, expression of Kup_DMR_40800_ in TK2446 results in growth on low potassium.

These findings suggest that Kup_DMR_40800_ has the ability to transport potassium. In *E. coli*, Kup spans the inner membrane and transports potassium ions into the cytoplasm. General import of potassium into the cell cannot be the only function of Kup_DMR_40800_, as the expression of a second copy of Kup_DMR_15830_ was not able to complement the magnetic phenotype of *kup* allele 1 RS-1. Kup_DMR_15830_ is functional for potassium transport, as it was also able to rescue TK2446 growth. Whether Kup_DMR_40800_ is transporting potassium at a special location or time for magnetosome synthesis, or whether it plays an entirely different role while retaining some potassium transport capability remains to be determined.

## Discussion

Magnetotactic bacteria are a diverse group but our mechanistic understanding of magnetosome synthesis is based only on the model α-proteobacteria. Relying on these model systems fails to elucidate the divergent magnetosome phenotypes of RS-1 and other non-α-proteobacterial magnetotactic bacteria. RS-1 is well placed to expand our understanding of magnetosome synthesis because it is a representative of a larger group of δ-proteobacteria, which produce bullet-shaped magnetite crystals.

As some of the *mam* genes are conserved among all sequenced members of magnetotactic bacteria, it appears that magnetosome formation evolved once. Whether the most recent common ancestor of all Proteobacteria, Nitrospirae and the OP3 division was a magnetotactic bacterium, or whether these genes moved through horizontal gene transfer remains under debate [14]. Within the most deeply branching clades of magnetotactic bacteria, including the one containing RS-1, bacteria synthesize bullet-shaped crystals, suggesting this is an ancestral form of magnetosome synthesis [14]. By studying this process, as we have undertaken here, we can increase our understanding of the diversity of the genes and mechanisms behind magnetosome formation, as well as begin to understand its origins.

### Saturation and limitations of our screen

In this study we isolated 26 single-gene mutants of RS-1 with magnetic phenotypes. The causative mutation for each mutant was identified with whole genome sequencing and confirmed with complementation analysis. We found mutations in 10 genes so that, on average, 2.6 alleles were isolated per mutated gene. Using the Poisson distribution, we calculated that with a frequency of 2.6 mutations per genetic locus there is probability of 0.074 that other viable targets have not been found in our screen. Therefore, within the constraints of our genetic strategy, 92.6% of the non-essential genes that could produce the desired phenotype when disrupted have been identified.

In addition to the screen not being fully saturated, there are several reasons that it could have failed to identify genes important for the magnetic phenotype of RS-1. For example, mutations in essential genes would not be identified by this method. Although the entire MAI is dispensable for growth under laboratory conditions, one could imagine a situation where the loss of one gene, but not the whole MAI, resulted in toxic magnetosome intermediates and a growth defect, though this has not been identified in the genetic dissections of the α-proteobacterial MAIs. Even if the growth defect of a mutant were slight, the multiple rounds of selection and outgrowth used here ensure that it could not be isolated. In addition to growth defects, genetic redundancy and the potentially reduced susceptibility of some loci to mutation could prevent the identification of mutants with interesting phenotypes. There is reason to suspect that at least a couple genes are missing from this screen. *mamI* (amb0962), a gene essential for membrane remodeling in AMB-1 [29], was not identified in this study. However, several *mamI* homologs have been identified in RS-1 (*mamI-1*, DMR_41140, DMR_41040) [11], suggesting that there could be redundancy among the RS-1 *mamI* genes. *mamM* (*DMR_41020*), *mamO* (*DMR_41070*), and *mamE* (*DMR_41080*) are additional genes that play important roles in the α-proteobacteria but whose RS-1 homologs were not mutated in our screen. In addition, one or more factors present in groups III and IV that are required for RS-1 magnetosome synthesis are clearly missing from this study, as deletion 3 could not be complemented by *tauE* alone.

The mutagenesis dosage used in this screen (50% cell survival) resulted in some mutants with only one change from WT and others with dozens of changes. Because the location of a mutated gene within the MAI could be used in this case to pick the candidate causative mutation, the high number of changes in some mutants was not a problem. If no such information is available to help identify candidates, lower doses of mutagenesis might result in individuals with fewer mutations. Alternatively, candidate causative mutations could be identified as those shared by a number of individuals with similar phenotypes and many mutations. Such a strategy has been pursued to identify attenuated mutants of the obligate intracellular pathogen *Chlamydia trachomatis* [47].

### Fewer Magnetic Particles (*fmp*) genes

Two genes identified in this screen, *DMR_41090* and *DMR_41100*, had previously been named based on their homology to other magnetosome genes. *DMR_41090* encodes a protein consisting of three PDZ domains, a protein-protein interaction domain found in magnetosome proteins including MamP and MamE. *DMR_41090* was originally annotated *mamP* (amb0970) [17], however DMR_41090 lacks the heme-binding domain that all other MamP proteins have. More recently it was suggested that *DMR_41090* be re-annotated *mamE-Cter* [11], a name that does not follow the conventional nomenclature for bacterial genes. As PDZ domains are not a unique feature of MamP and MamE proteins, we have renamed *DMR_41090 fmpA* for Fewer Magnetic Particles A.

Based on sequence, DMR_41100 has two domains with redox activity, an amino-terminal magnetochrome domain and a carboxy-terminal NifX/NifB domain. NifX/NifB domains are involved in the synthesis of the iron molybdenum cofactor, and bind redox-active iron-sulfur clusters. Due to its magnetochrome domain, *DMR_41100* was originally annotated *mamT* (amb0976) [17], but reannotated *mamP-like* because it also contains a PDZ domain [11]. However, these features are not unique to MamP and are also found in combination in α-proteobacterial MamEs. NifX/NifB is a motif not found in other magnetosome proteins. For these reasons we have renamed *DMR_41100 fmpB* for Fewer Magnetic Particles B.

### Magnetosome formation in RS-1 and its implications for all magnetotactic bacteria

In this work we identify for the first time a list of genes that are required for the formation of magnetosomes in a magnetotactic δ-proteobacterium. In addition to some of the anticipated homologs of *mam* genes, we discovered a number of genes without direct homologs in the α-proteobacteria. When mutated, *mad1*, *mad2*, and *mad6* as well as *fmpA* and *fmpB*, had less severe phenotypes than many of the *mam* homologs. Indeed, mutations in genes that RS-1 and the α-proteobacteria share, such as *mamE*, *mamB*, *mamM* and others described in Figure 1C, have more severe phenotypes in the α-proteobacteria than those in genes that RS-1 lacks, such as *mamS* or *mamT*, whose mutants in the α-proteobacteria are able to synthesize limited magnetite particles [20], [29], [30], [48]. Perhaps, as bioinformatics surveys of magnetosome genes have suggested [49], all magnetotactic bacteria share the most fundamental and important genes, or the genes required for taking the first steps in magnetosome synthesis, but then each has developed its own set of accessory genes to complete the process as best suited to its own needs.

It is surprising that some ubiquitous bacterial transporters, such as those encoded by *kup* and *tauE*, are required for magnetosome formation in RS-1. Almost nothing is understood about *tauE*, previously known as DUF81. TauE consists of transmembrane helices, consistent with a transporter. Although DUF81 is widely distributed among bacteria, only two have been studied. TsaS (AAT81376), from *Comamonas testosteroni*, was hypothesized to be an importer of 4-toluenesulfonate across the inner membrane based on its sequence and organization in an operon involved in 4-toluenesulfonate catabolism [50]. TauE (YP_841384), from *Cupriavidus necator*, which lends the DUF81 group its name, was hypothesized to export sulfite from the cell to remove the byproduct of various sulfonate metabolisms [44]. In addition to sequence analysis and operon organization, the expression of *tauE_C. necator_* was measured and shown to be dependent on growth with sulfonate. The non-magnetic phenotype of *tauE*_RS-1_ mutants shown here is the first example of a phenotype for a gene encoding only a DUF81 domain. Studies of TauE*_C. necator_* and TsaS suggest that TauE_RS-1_ may function as an anion transporter. What it transports, in which direction, and why it is also found fused to the protease domains of MamE and MamO proteins all remain to be determined.

**Figure 10:**
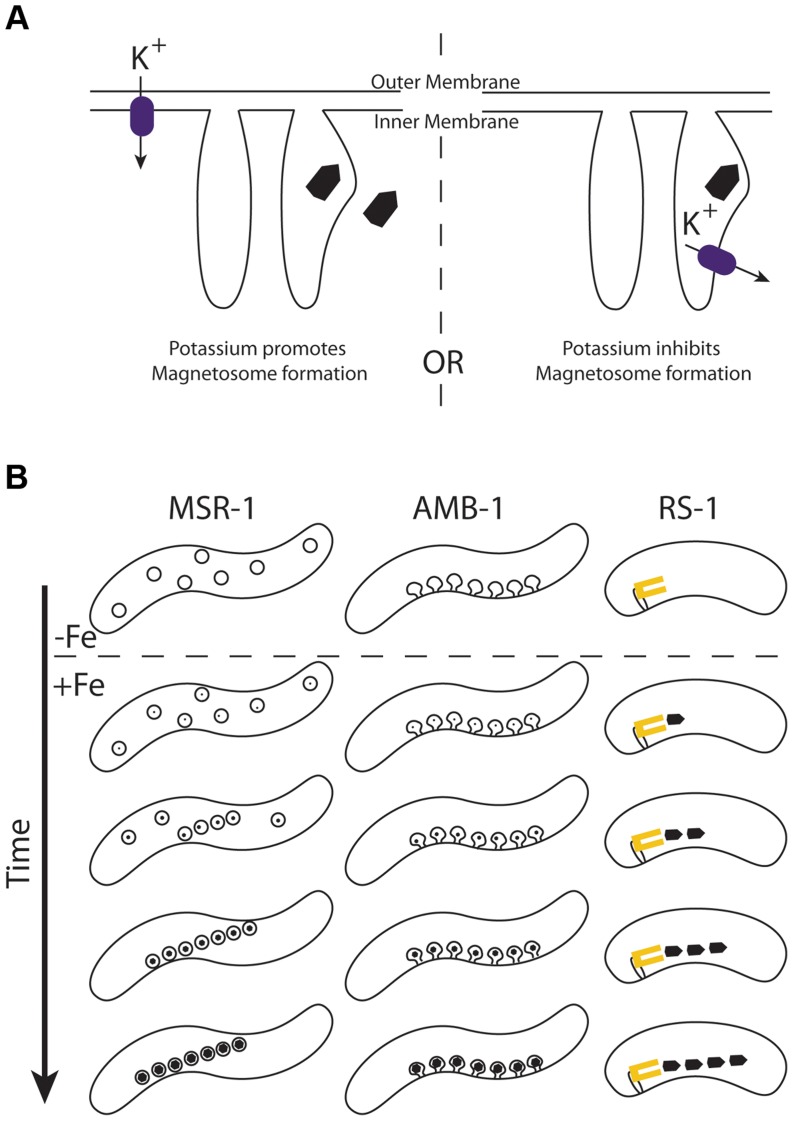
A) Models for how potassium effects magnetosome formation. Kup (purple rectangle) may transport potassium into the cell through the inner membrane (left) or out of the magnetosome through a hypothetical magnetosome membrane (right). In the former case, potassium would be beneficial to magnetosome formation, and the magnetite crystal may be built inside or outside a membrane. In the latter case potassium would inhibit magnetosome formation that occurs within a magnetosome membrane. B) Proposed model for magnetosome synthesis in RS-1. The α-proteobacteria MSR-1 and AMB-1 produce many crystals at once as each preexisting magnetosome membrane represents a potential site of synthesis. In AMB-1 these membranes are linearly arranged invaginations of the inner membrane [18]. In MSR-1 these membranes are vesicles that become linearly arranged with the guidance of the magnetic fields of the growing crystals [42]. In RS-1 we suggest that magnetite crystals are produced one at a time from a single magnetosome factory that is associated with the membrane (yellow rectangle).

Like TauE, Kup is a widely distributed bacterial transporter. Although its genetic role in potassium transport is well studied in *E. coli*, there is still little mechanistic understanding of Kup function that could help us distinguish what makes Kup_DMR_40800_ different from Kup_DMR_15830_. Though we present evidence in this work that Kup_DMR_40800_ can transport potassium when expressed in *E. coli*, we don't know if potassium transport is its function in magnetosome synthesis. Because Kup transports potassium from the periplasmic space to the cytoplasm, one can imagine it either bringing potassium into the cell (as it does in *E. coli*), or if localized to a hypothetical magnetosome membrane, clearing potassium from the magnetosome space. These two possibilities are described in Figure 10A. In the first case, potassium is required for magnetosome synthesis, and in the second case it is a contaminant that must be removed.

Potassium has been implicated in the biomineralization of teeth, where it is hypothesized to activate important enzymes, serve as a counter-ion to the negatively charged extra-cellular matrix, and help regulate the coordinated activation of the matrix along the mineralization front for calcium deposition [51]. However, magnetite biomineralization in magnetotactic bacteria, unlike the biomineralization of bones and teeth, is an intracellular process that is not thought to involve an organic matrix [52], so it is difficult to imagine potassium playing a similar role in RS-1.

Finally, a number of our mutants had intermediate phenotypes, with magnetic responses below WT levels. This is also found for a number of genes in the α-proteobacteria. These biomineralization mutants in AMB-1 and MSR-1 have full chains of crystals like WT, but each crystal is either too small or the wrong shape [20], [29], [30], [48]. We originally expected RS-1 biomineralization mutants would have similar phenotypes, with full chains of poorly synthesized crystals. However, without exception RS-1 mutants with a magnetic response contained WT-like crystals in much fewer number than the WT. One explanation for this discrepancy is that such α-proteobacterial style mutants exist but were not identified in this screen because the isolated mutants had to be able to flow through a magnetic column, so only strains where some individual cells contained no magnetic particles could be isolated. However, another possibility is that this is due to a fundamental difference between how the α- and δ-proteobacteria synthesize magnetosomes.

When the α-proteobacteria are reintroduced to iron after iron starvation, they synthesize a full chain of crystals all at once, first possessing many small crystals, then many large crystals [18]. However, when RS-1 is reintroduced to iron after iron starvation, cells first possess one, then two or three full-sized crystals, with more at later time points [22]. Even in the absence of iron, AMB-1 is known to contain empty magnetosome membranes [18], each poised to mineralize its own crystal. Perhaps RS-1 crystals are made one at a time from a central magnetosome factory. In the case of biomineralization mutants, the chain of α-proteobacterial membranes will each try to build a crystal with some inefficiency, resulting in a full chain of small crystals. In an RS-1 biomineralization mutant, the central factory is the only point of synthesis, so the inefficiency stemming from the mutation results in many fewer crystals produced. The dissociation of magnetosome crystals from their point of synthesis could also explain why many magnetosome proteins in RS-1 are integral membrane proteins, but no membranes have been observed around magnetosome crystals. In this model, the magnetosome factory would be imbedded in a membrane, but mature crystals would be free to move away from this membrane after synthesis. This new model for δ-proteobacterial magnetosome synthesis is described in Figure 10B.

### Modern sequencing tools and the new accessibility of interesting biological diversity

In this work we applied classical mutagenesis combined with modern sequencing to query a previously opaque system: the magnetic δ-proteobacteria. A genetic analysis of these bacteria was not previously possible, as the genetic tools that allow the construction of targeted deletions or the mapping of mutations are not accessible for bacteria with low rates of transformation. With the minimal genetic tools available for RS-1 we were also able to complement the mutations, the gold standard in genetics for demonstrating that a mutation in a gene is responsible for a phenotype. This strategy confirmed and extended our understanding of the genetic and molecular mechanisms at play in magnetotactic bacteria as understood from the α-proteobacterial model systems, suggesting roles for unexpected proteins such as Kup, and reemphasizing the importance of overlooked domains, such as the TauE domains fused to MamO and MamE proteins. We have also discovered phenotypes for some *mad* genes, which had previously only been tied to magnetosome synthesis through bioinformatics analysis.

These insights have been possible because of the depth of understanding of the model α-proteobacteria combined with the interesting differences between these bacteria and RS-1. Based on our success and the success of similar genetic analyses with the even more recalcitrant *Chlamydia trachomatis* [47], we envision that the strategy outlined here can be applied to any culturable organism to make and identify mutations of interest. With this and other high-throughput genetic tools that are emerging, such as Tn-seq [53], diverse and obscure systems like the magnetotactic bacteria are sure to be fertile ground for new genetic and mechanistic insights.

## Materials and Methods

### Strains

Cloning was performed in *E. coli* DH5α *λ-pir*. Matings, described below, were performed with *E. coli* WM3064 [54]. Potassium growth experiments, described below, were performed with *E. coli* TK2446 (*F^−^ thi rha lacZ nagA* Δ(*kdp FAB*)*5 trkD1 trkG*(*kan*) *trkH*(*cam*) Δ(*trkA-mscL*), gift of Ian Booth). The WT RS-1 used in this work (AK80) is a spontaneous mutant of the ATTC RS-1 (AK8) that was isolated in our lab. Unlike AK8, AK80 is non-motile and does not make biofilms, allowing us to take C_mag_ measurements more easily. The differences in AK8 and AK80 from the published RS-1 genome [12] are listed in Table S5. RS-1 was grown in RS-1 Growth Media as described previously [22] except that 12 mM Hepes buffer was included in the media and the pH set to 6.7 with sodium hydroxide. For RS-1 carrying a plasmid, 125 ug/mL kanamycin sulfate was added to the media.

### Mutagenesis

Wild type RS-1 was passaged two times in liquid without supplied iron. Cells were mutagenized either with 10 mJ of ultraviolet radiation or with 30% ethyl methane sulfonate for one hour. Both treatments resulted in about 50% survival. Table S6 indicates which mutants resulted from which treatment. After mutagenesis, cells were grown in liquid in the presence of iron. The resultant cultures were passed over a magnetized MACS MS column (Miltenyi Biotec), and the flow-through was inoculated into fresh liquid media. This outgrowth and enrichment was performed four times. By the last enrichment, most cells were non-magnetic and were found in the flow-through of the column, which was diluted and plated for single colonies. The resultant colonies were screened by PCR for loss of the magnetosome island (see below). Those colonies that had a magnetic phenotype and possessed the island were saved for further analysis.

### C_mag_ assays

C_mag_ assays were performed as previously described [18], except that the optical density was measured at 650 nm. For strains carrying a plasmid, kanamycin (which is in the form of kanamycin sulfate) was not included in the culture to be measured for C_mag_, as sulfate reduction interferes with magnetosome formation in RS-1 [22].

### Electron microscopy

Whole-cell transmission electron microscopy and high-resolution transmission electron microscopy were performed as previously described [22]. Cryo-ultramicrotomy was performed as previously described [18].

### Sequencing

Genomic DNA was isolated from 10 mL of RS-1 culture with the Qiagen Blood and Tissue kit. Library prep was performed by the Functional Genomics Laboratory at the University of California, Berkeley by following the standard Illumina-compatible library preparation protocol by IntegenX (now Wafergen). Samples were fragmented using the Covaris S220 to a target insert size of 250 bps and sample fragmentation length was confirmed using an Agilent Bioanalyzer. Samples were then loaded on the IntegenX Apollo 324 system. IntegenX PrepX Library kits were used to undergo end-repair, A-tail addition, adapter ligation, and size selection using AMPure XP beads. Samples were quantified using the Qubit and PCR amplified to incorporate indexes and flow cell binding regions. Final libraries were quantified using the Qubit, Bioanalzyer and qPCR. The indexed libraries were combined up to 35 per lane then sequenced with a 50 base pair, single-end run on a HiSeq2000 instrument using V3 chemistry and standard Illumina analysis software (RTA/Casava). Reads were aligned to the reference genome (NCBI Reference Sequence: NC_012796.1) with the BWA aligner [55], and single nucleotide polymorphisms were called with Freebayes [56]. Deletions and insertions were visualized with the Integrative Genomics Viewer [57]. Whole genome coverage was visualized with Qualimap [58]. Sequencing data is available on the NCBI Sequence Read Archive accession number SRP045907.

### PCR genotyping of mutants

PCR and agarose gel electrophoresis or Sanger sequencing were used to check for the presence of the MAI and to confirm mutations identified by whole-genome sequencing. Primers are listed in Table S7. In the case of the MAI test, two PCR products of varying sizes, one inside and one outside the island, were produced together in one PCR reaction then their presence or absence visualized on a gel. To confirm the large deletions, PCR was performed spanning the deletion scar so that a PCR product was possible only in the case of the putative deletion. To test for putative transposon insertions, a difference in size between the WT and mutant PCR products of each gene was observed. To confirm point mutations, the candidate gene was amplified by PCR then sequenced with Sanger sequencing.

### Multiple sequence alignments

MamB homologs from non-magnetotactic bacteria were identified with BLAST [59]. MamB, MamL, Kup sequences, and the 16S sequences used in Figure 1A were aligned with Clustal Omega [60]. Alignments were visualized with JalView [61].

### Plasmids and cloning

For a list of plasmids used in this work, see Table S4. Cloning was performed using the Gibson method [62]. pBMK7 [28] was digested with HindIII and SalI to create pLR6 and with SalI to create the remaining pBMK7-based plasmids. pLR6 was digested with SalI to create the pLR6-based plasmids. pMscSH6 [63] was digested with NcoI and XhoI to create the pMscSH6-based plasmids. All inserts were amplified with the indicated primers from RS-1 genomic DNA except for *kup_E. coli_*, which was amplified from *E. coli* DH5α cells, and *gfp*, which was amplified from pAK22 [64].

### Transconjugation

RS-1 was transformed by conjugation with *E. coli* strain WM3064. WM3064 cells carrying the plasmid to be transformed into RS-1 were grown overnight in Luria Broth with 50 ug/mL kanamycin sulfate and 0.3 mM DAP. 500 uL of WM3064 culture was washed once in Luria Broth then combined with 3 to 10 mL of RS-1 culture in 25 uL RS-1 Growth Media with 0.3 mM DAP for 4 to 6 hours. The cells were plated on RS-1 Growth Media Agar with kanamycin.

### Magnetic field imaging using nitrogen-vacancy centers in diamond

The magnetic fields produced by wild type and *mad 1* mutant RS-1 bacteria were measured using nitrogen-vacancy (NV) color centers in diamond [43]. This magnetic imaging technique employed a 2.5×2.5×0.5 mm diamond chip produced using chemical vapor deposition, and engineered to contain a dense layer of NV centers at the diamond surface (surface density ∼3×10^11^ NV/cm^2^, depth ∼20 nm). Bacteria were placed on this diamond surface and allowed to dry. The magnetic fields sensed by the NV centers in the vicinity of each bacteria were then measured using optically detected magnetic resonance (ODMR) spectroscopy [43], where the magnetic field is determined from the NV electronic spin-flip resonance frequencies by monitoring the spin-state dependent fluorescence signal from the NV centers while simultaneously applying laser excitation and a microwave field with variable frequency. By imaging the fluorescence from the NV centers onto a sCMOS camera, images of the magnetic field at the diamond surface were recorded with sub-micron resolution over 400 µm fields of view. These magnetic images were co-registered with optical transmission images of the bacteria on the diamond surface using the same camera.

For one set of measurements, all three vector components of the magnetic field at the diamond surface were determined by independently measuring the magnetic field projections along each of four possible NV crystallographic orientations, in the presence of a 1.2 mT bias field produced by a set of Helmholtz coils. The absolute magnitude of the magnetic field signal in the vicinity of each bacterium within a field of view was then computed and used to estimate the relative magnetic moments within the sampled wild type and *mad 1* mutant RS-1 bacteria populations, and also to estimate the average magnetic moment of the wild type RS-1 bacteria by comparing the signals with previous measurements on AMB-1 bacteria [43]. In another set of measurements, only one projection of the magnetic field vector was measured across the diamond surface, along a single NV crystallographic direction while applying a uniform 20.5 mT bias field along the same direction using a pair of permanent magnets. The two sets of measurements were conducted on the same bacteria within a given field of view to determine whether or not the magnetic signal near each bacterium changed as a function of the magnitude of the uniform bias field.

### Potassium growth experiments

*E. coli* TK2446 carrying various plasmids were grown in LK medium (10 g tryptone, 5 g yeast extract, and 6.4 g KCl in 1 L) with 25 ug/mL carbenicillin. To test for growth on low potassium, cells from LK starter cultures were normalized for optical density, washed three times with K_0_ buffer [65] (16.47 g Na_2_HPO_4_*12 H_2_O, 3.13 g NaH_2_PO_4_*2 H_2_O, and 1.05 g (NH_4_)_2_SO_4_ in 1 L), then plated on K_15_ or K_120_ plates (15 g agar autoclaved in 800 mL water, then 200 mL 5X K_0_ buffer added, supplemented with 0.2% glucose, 0.0001% thiamine, 0.4 mM MgSO4, and 6 uM iron and 15 or 120 mM potassium chloride) with 12.5 ug/mL carbenicillin and 0.4 mM IPTG as 10 ul drops of 1∶10 serial dilution series.

## Supporting Information

S1 Fig Coverage across the genome for WT and an MAI deletion strain.
(TIF)

S2 Fig TEM of WT and mutant cells isolated in the screen. Scale bar 200 nm for whole cells and 100 nm for insets.
(TIF)

S1 Table The position of and features missing from the large deletions.
(DOCX)

S2 Table Additional mutations in each mutant.
(DOCX)

S3 Table Plasmid carried, replicate number, and C_mag_ value for each strain in Figure 5.
(DOCX)

S4 Table Plasmids used in this study.
(DOCX)

S5 Table Mutations identified in RS-1 Wild Type strains.
(DOCX)

S6 Table Mutagen used for each mutant.
(DOCX)

S7 Table Primers used in this study.
(DOCX)
