# Supplementary figures and images for: A Genetic Strategy for Probing the Functional Diversity of Magnetosome Formation
Source: PLoS Genet. 2015 Jan 8;11(1):e1004811. doi: 10.1371/journal.pgen.1004811 (PMC4287615; doi:10.1371/journal.pgen.1004811)

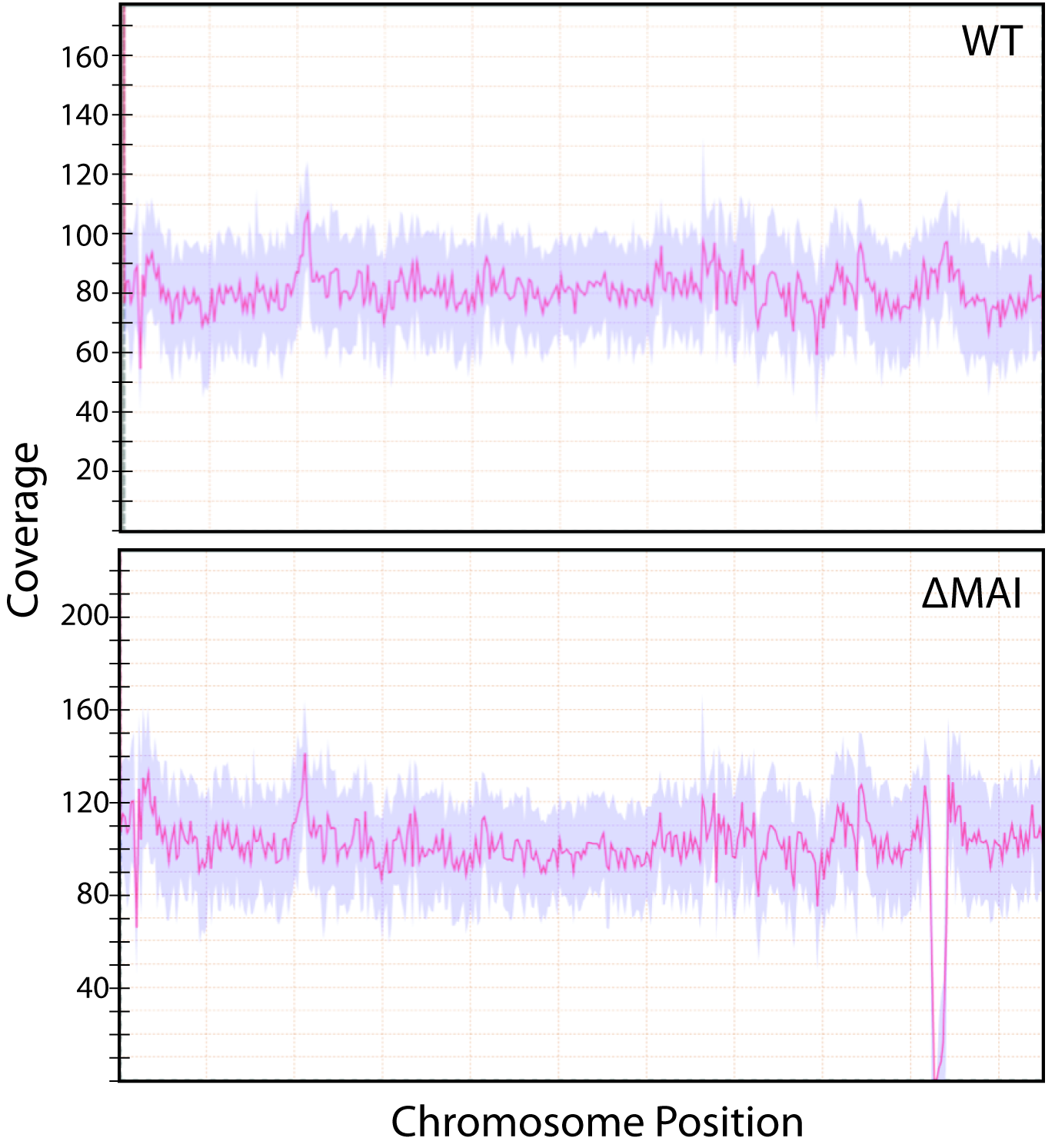

Supplement: S1 Fig — Coverage across the genome for WT and an MAI deletion strain. (TIF) [file pgen.1004811.s001.tif]

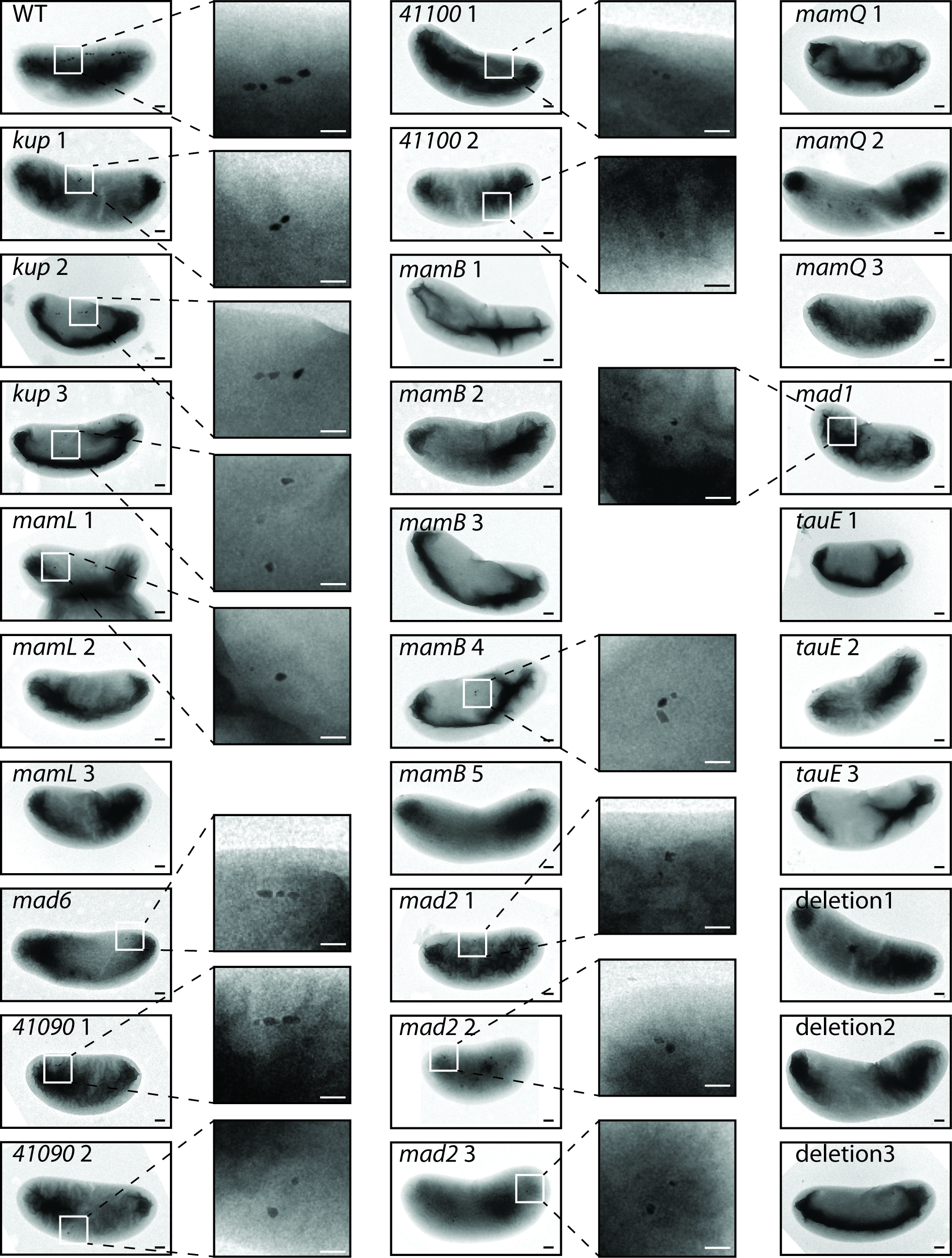

Supplement: S2 Fig — TEM of WT and mutant cells isolated in the screen. Scale bar 200 nm for whole cells and 100 nm for insets. (TIF) [file pgen.1004811.s002.tif]
