# Supplementary material for: A Genetic Strategy for Probing the Functional Diversity of Magnetosome Formation
Source: PLoS Genet. 2015 Jan 8;11(1):e1004811. doi: 10.1371/journal.pgen.1004811 (PMC4287615; doi:10.1371/journal.pgen.1004811)
Supplement: S1 Table — The position of and features missing from the large deletions. (DOCX) [file pgen.1004811.s003.docx]

Table S1: the position and features missing from the large deletions

| **Large Deletion** | **Nucleotides Missing** | **Features Missing** | **Other changes in genome** |
| --- | --- | --- | --- |
| 1 | 4639461 to 4657224 | Group I from *mamM* to *mamI1* | 0 |
| 2 | 4656947 to 4665431 | All but last gene in group II | 2 |
| 3 | 4654946-4657225 and 4658876 to 4704248 | Groups II-IV | 3 |
