# Supplementary material for: A Genetic Strategy for Probing the Functional Diversity of Magnetosome Formation
Source: PLoS Genet. 2015 Jan 8;11(1):e1004811. doi: 10.1371/journal.pgen.1004811 (PMC4287615; doi:10.1371/journal.pgen.1004811)
Supplement: S2 Table — Additional mutations in each mutant. (DOCX) [file pgen.1004811.s004.docx]

Table S2: Additional mutations in each mutant

| **Gene** | **Allele** | **Number of changes** | **Nucleotide changes** |
| --- | --- | --- | --- |
| *kup* | 2 | 15 | C265712T |
|  |  |  | C319599T |
|  |  |  | C449606T |
|  |  |  | C849975T |
|  |  |  | C1122418T |
|  |  |  | C1165464T |
|  |  |  | C1275927T |
|  |  |  | G1718082A |
|  |  |  | C3984402T |
|  |  |  | C4454588T |
|  |  |  | C4466570T |
|  |  |  | C4487776T |
|  |  |  | C4621527T |
|  |  |  | G4830232A |
|  |  |  | G4957825A |
| *kup* | 3 | 2 | T1533769A |
|  |  |  | C3984402T |
| *mamL* | 1 | 3 | C13412T |
|  |  |  | C97047T |
|  |  |  | C3984402T |
| *mamL* | 2 | 5 | T1533769A |
|  |  |  | C2760662T |
|  |  |  | A2879484G |
|  |  |  | C3984402T |
|  |  |  | T5099594A |
| *mad6* | 1 | 1 | C3984402T |
| *fmpA* | 1 | 3 | C2196437T |
|  |  |  | G3896689T |
|  |  |  | C3984402T |
| *fmpA* | 2 | 1 | C3984402T |
| *fmpB* | 1 | 1 | C3984402T |
| *fmpB* | 2 | 2 | G3675475T |
|  |  |  | C3984402T |
| *mamB* | 2 | 2 | C3984402T |
|  |  |  | G5184605A |
| *mamB* | 3 | 1 | G4722396A |
| *mamB* | 4 | 20 | G405211A |
|  |  |  | G1605434A |
|  |  |  | G1895970A |
|  |  |  | G1969235A |
|  |  |  | G2055954A |
|  |  |  | A2088209A |
|  |  |  | G2139952A |
|  |  |  | G2153085A |
|  |  |  | C2214879T |
|  |  |  | C2498452T |
|  |  |  | C2795620T |
|  |  |  | C2899577T |
|  |  |  | C3130325T |
|  |  |  | C3199115T |
|  |  |  | C3204828T |
|  |  |  | C3223498T |
|  |  |  | C3667394T |
|  |  |  | C3984402T |
|  |  |  | C4745062T |
|  |  |  | C5115109T |
| *mamB* | 5 | 8 | C3984402T |
|  |  |  | C4222699T |
|  |  |  | G4422305A |
|  |  |  | C4544045T |
|  |  |  | C4797563T |
|  |  |  | C4937078T |
|  |  |  | G5022729A |
|  |  |  | G5026418A |
| *mad2* | 1 | 12 | C89463T |
|  |  |  | C274830T |
|  |  |  | C296803T |
|  |  |  | G612712A |
|  |  |  | C997993T |
|  |  |  | C1035611T |
|  |  |  | A3389230G |
|  |  |  | C3984402T |
|  |  |  | G4681418A |
|  |  |  | G4743794A |
|  |  |  | G5157064A |
|  |  |  | G5236235A |
| *mad2* | 2 | 1 | C3984402T |
| *mad2* | 3 | 2 | C3984402T |
|  |  |  | G4709302A |
| *mamQ* | 1 | 23 | G530260A |
|  |  |  | C833913T |
|  |  |  | G1021790A |
|  |  |  | G1022878A |
|  |  |  | C1234017T |
|  |  |  | C1256645T |
|  |  |  | C1286248T |
|  |  |  | C1657496T |
|  |  |  | G1759749A |
|  |  |  | G1918966A |
|  |  |  | G2024140A |
|  |  |  | G2545944A |
|  |  |  | G2559550A |
|  |  |  | G2689837A |
|  |  |  | C3401267T |
|  |  |  | C3984402T |
|  |  |  | C4279433T |
|  |  |  | G4536793A |
|  |  |  | G4585609A |
|  |  |  | G4709338A |
|  |  |  | G4767732A |
|  |  |  | C4779302T |
|  |  |  | C4779570T |
| *mamQ* | 2 | 6 | C3984402T |
|  |  |  | C4002325T |
|  |  |  | C4018399T |
|  |  |  | G4702101A |
|  |  |  | G4731348A |
|  |  |  | G4754874A |
| *mamQ* | 3 | 1 | C3984402T |
| *mad1* | 1 | 1 | C3984402T |
| *tauE* | 1 | 1 | C3984402T |
| *tauE* | 2 | 8 | C3984402T |
|  |  |  | G4017400A |
|  |  |  | C4078504T |
|  |  |  | G4163502A |
|  |  |  | C4508910T |
|  |  |  | C4589293T |
|  |  |  | C4767932T |
|  |  |  | C5200510T |
| *tauE* | 3 | 2 | C3984402T |
|  |  |  | T3155009A |
| *Deletion 2* |  | 2 | C3908387T |
|  |  |  | A5014288C |
| *Deletion 3* |  | 3 | C50600T |
|  |  |  | C3984402T |
|  |  |  | G4032980A |
