# Supplementary material for: A Genetic Strategy for Probing the Functional Diversity of Magnetosome Formation
Source: PLoS Genet. 2015 Jan 8;11(1):e1004811. doi: 10.1371/journal.pgen.1004811 (PMC4287615; doi:10.1371/journal.pgen.1004811)
Supplement: S3 Table — Plasmid carried, replicate number, and Cmag value for each strain in Figure 5. (DOCX) [file pgen.1004811.s005.docx]

Table S3: Plasmid carried, replicate number, and C_mag_ value for each strain in Figure 5

| **Strain** | **Plasmid** | **n** | **C_mag_** | **Standard Deviation** |
| --- | --- | --- | --- | --- |
| WT | pBMK7 | 78 | 1.484 | 0.115 |
| *kup1* control | pBMK7 | 6 | 1.000 | 0.000 |
| *kup2* control | pBMK7 | 6 | 1.000 | 0.000 |
| *kup3* control | pBMK7 | 6 | 1.000 | 0.000 |
| *mamL1* control | pBMK7 | 5 | 1.000 | 0.000 |
| *mamL2* control | pBMK7 | 5 | 1.000 | 0.000 |
| *mamL3* control | pBMK7 | 6 | 1.000 | 0.000 |
| *mad6* control | pBMK7 | 5 | 1.036 | 0.044 |
| *fmpA1* control | pBMK7 | 5 | 1.038 | 0.015 |
| *fmpA2* control | pBMK7 | 6 | 1.000 | 0.000 |
| *fmpB1* control | pBMK7 | 5 | 1.078 | 0.024 |
| *fmpB2* control | pBMK7 | 6 | 1.011 | 0.007 |
| *mamB1* control | pBMK7 | 5 | 1.000 | 0.000 |
| *mamB2* control | pBMK7 | 6 | 1.000 | 0.000 |
| *mamB3* control | pBMK7 | 4 | 1.000 | 0.000 |
| *mamB4* control | pBMK7 | 5 | 1.018 | 0.004 |
| *mamB5* control | pBMK7 | 6 | 1.000 | 0.000 |
| *mad2_1* control | pBMK7 | 5 | 1.000 | 0.000 |
| *mad2_2* control | pBMK7 | 6 | 1.000 | 0.000 |
| *mad2_3* control | pBMK7 | 6 | 1.000 | 0.000 |
| *mamQ1* control | pBMK7 | 6 | 1.000 | 0.000 |
| *mamQ2* control | pBMK7 | 6 | 1.000 | 0.000 |
| *mamQ3* control | pBMK7 | 6 | 1.000 | 0.000 |
| *mad1_1* control | pBMK7 | 2 | 1.000 | 0.000 |
| *tauE1* control | pBMK7 | 5 | 1.000 | 0.000 |
| *tauE2* control | pBMK7 | 6 | 1.000 | 0.000 |
| *tauE3* control | pBMK7 | 6 | 1.000 | 0.000 |
| Deletion 2 control | pBMK7 | 6 | 1.000 | 0.000 |
| Deletion 3 control | pBMK7 | 5 | 1.000 | 0.000 |
| *kup1* complemented | pLR41 | 6 | 1.201 | 0.052 |
| *kup2* complemented | pLR41 | 5 | 1.188 | 0.071 |
| *kup3* complemented | pLR41 | 5 | 1.233 | 0.063 |
| *mamL1* complemented | pLR67 | 5 | 1.023 | 0.023 |
| *mamL2* complemented | pLR67 | 5 | 1.282 | 0.065 |
| *mamL3* complemented | pLR67 | 6 | 1.174 | 0.063 |
| *mad6* complemented | pLR60 | 6 | 1.208 | 0.088 |
| *fmpA1* complemented | pLR61 | 6 | 1.224 | 0.051 |
| *fmpA2* complemented | pLR61 | 6 | 1.015 | 0.009 |
| *fmpB1* complemented | pLR62 | 5 | 1.191 | 0.030 |
| *fmpB2* complemented | pLR62 | 6 | 1.056 | 0.016 |
| *mamB1* complemented | pLR20 | 5 | 1.008 | 0.005 |
| *mamB2* complemented | pLR20 | 5 | 1.143 | 0.088 |
| *mamB3* complemented | pLR20 | 5 | 1.170 | 0.034 |
| *mamB4* complemented | pLR20 | 6 | 1.324 | 0.021 |
| *mamB5* complemented | pLR20 | 6 | 1.433 | 0.057 |
| *mad2_1* complemented | pLR63 | 6 | 1.259 | 0.056 |
| *mad2_2* complemented | pLR63 | 5 | 1.332 | 0.049 |
| *mad2_3* complemented | pLR63 | 6 | 1.389 | 0.088 |
| *mamQ1* complemented | pLR68 | 6 | 1.104 | 0.022 |
| *mamQ2* complemented | pLR68 | 5 | 1.242 | 0.054 |
| *mamQ3* complemented | pLR68 | 6 | 1.214 | 0.028 |
| *mad1_1* complemented | pLR65 | 5 | 1.164 | 0.044 |
| *tauE1* complemented | pLR56 | 6 | 1.274 | 0.044 |
| *tauE2* complemented | pLR56 | 6 | 1.204 | 0.038 |
| *tauE3* complemented | pLR56 | 6 | 1.283 | 0.052 |
| Deletion 2 complemented | pLR56 | 5 | 1.194 | 0.029 |
| Deletion 3 complemented | pLR56 | 6 | 1.003 | 0.008 |
