# Supplementary material for: A Genetic Strategy for Probing the Functional Diversity of Magnetosome Formation
Source: PLoS Genet. 2015 Jan 8;11(1):e1004811. doi: 10.1371/journal.pgen.1004811 (PMC4287615; doi:10.1371/journal.pgen.1004811)
Supplement: S4 Table — Plasmids used in this study. (DOCX) [file pgen.1004811.s006.docx]

Table S4: Plasmids used in this study

| **Plasmid name** | **Description** | **Source/derivation** | **Construction** |
| --- | --- | --- | --- |
| pAK22 | AMB-1 MamK-GFP | [64] | NA |
| pBMK7 | *Desulfovibrio* vector | [28] | NA |
| pMscSH6 | pTRC99A-based *E. coli* expression vector | [63] | NA |
| pLR6 | Expression from P_mamA_ in RS-1 | P_mamA_ cloned into pBMK7 | LRL29/LRL30 |
| pLR20 | Complementation of *mamB* | *mamB* cloned into pLR6 | LRL131/LRL132 |
| pLR41 | Complementation of *kup* | *kup* cloned into pLR6 | LRL159/LRL160 |
| pLR50 | *E. coli* expression of *kup_DMR_40800_* | *kup_DMR_41800_* cloned into pMscSH6 | LRL186/LRL187 |
| pLR52 | *E. coli* expression of *kup_E.coli_* | *kup_E.coli_* cloned into pMscSH6 | LRL190/LRL191 |
| pLR56 | Complementation of *tauE* | P_tauE_-*tauE* cloned into pBMK7 | LRL177/LRL128 |
| pLR58 | *E. coli* expression of GFP | *GFPmut2* cloned into pMscSH6 | LRL204/LRL205 |
| pLR60 | Complementation of *mad6* | *mad6* cloned into pLR6 | LRL208/LRL209 |
| pLR61 | Complementation of *lmrA* | *lmrA* cloned into pLR6 | LRL210/LRL211 |
| pLR62 | Complementation of *lmrB* | *lmrB* cloned into pLR6 | LRL212/LRL213 |
| pLR63 | Complementation of *mad2* | *mad2* cloned into pLR6 | LRL214/LRL215 |
| pLR65 | Complementation of *mad1* | *mad1* cloned into pLR6 | LRL218/LRL220 |
| pLR67 | Complementation of *mamL* | P_mamL_-*mamL* cloned into pBMK7 | LRL224/LRL207 |
| pLR68 | Complementation of *mamQ* | P_mamQ_-*mamQ* cloned into pBMK7 | LRL225/LRL217 |
