# Supplementary material for: A Genetic Strategy for Probing the Functional Diversity of Magnetosome Formation
Source: PLoS Genet. 2015 Jan 8;11(1):e1004811. doi: 10.1371/journal.pgen.1004811 (PMC4287615; doi:10.1371/journal.pgen.1004811)
Supplement: S5 Table — Mutations identified in RS-1 Wild Type strains. (DOCX) [file pgen.1004811.s007.docx]

Table S5: Mutations identified in RS-1 Wild Type strains

| **Strain** | **Gene Number** | **Predicted Function** | **Nucleotide change** | **Amino acid change** |
| --- | --- | --- | --- | --- |
| AK8 and AK80 | DMR_10250 | Hypothetical protein | T1141833A | Silent |
|  | DMR_20200 | Sensor histidine kinase | A2275924G | S414P |
|  | DMR_22150 | Amidophosphoribosyltransferase | T2490828C | D124G |
|  | DMR_32650 | Hypothetical protein | C3667394T | E225K |
|  | intergenic | NA | 4896303 removed | NA |
| AK80 | DMR_18030 | Molybdopterin oxidoreductase iron-sulfur cluster-binding subunit | 2012106 removed | Frameshift at position 55 |
|  | DMR_26110 | GGDEF domain protein | G2920008T | Premature stop at position 31 |
