# Supplementary material for: A Genetic Strategy for Probing the Functional Diversity of Magnetosome Formation
Source: PLoS Genet. 2015 Jan 8;11(1):e1004811. doi: 10.1371/journal.pgen.1004811 (PMC4287615; doi:10.1371/journal.pgen.1004811)
Supplement: S6 Table — Mutagen used for each mutant. (DOCX) [file pgen.1004811.s008.docx]

Table S6: Mutagen used for each mutant

| **Mutant** | **Mutagen** |
| --- | --- |
| *kup* 1 | UV |
| *kup* 2 | EMS |
| *kup* 3 | EMS |
| *mamL* 1 | EMS |
| *mamL* 2 | EMS |
| *mamL* 3 | EMS |
| *mad6* | EMS |
| *fmpA* 1 | EMS |
| *fmpA* 2 | EMS |
| *fmpB* 1 | EMS |
| *fmpB* 2 | EMS |
| *mamB* 1 | UV |
| *mamB* 2 | EMS |
| *mamB* 3 | EMS |
| *mamB* 4 | EMS |
| *mamB* 5 | EMS |
| *mad2* 1 | EMS |
| *mad2* 2 | EMS |
| *mad2* 3 | EMS |
| *mamQ* 1 | EMS |
| *mamQ* 2 | EMS |
| *mamQ* 3 | EMS |
| *mad1* | EMS |
| *tauE* 1 | UV |
| *tauE* 2 | EMS |
| *tauE* 3 | EMS |
| Deletion 1 | UV |
| Deletion 2 | UV |
| Deletion 3 | EMS |
| MAI | UV |
