# Supplementary material for: A Genetic Strategy for Probing the Functional Diversity of Magnetosome Formation
Source: PLoS Genet. 2015 Jan 8;11(1):e1004811. doi: 10.1371/journal.pgen.1004811 (PMC4287615; doi:10.1371/journal.pgen.1004811)
Supplement: S7 Table — Primers used in this study. (DOCX) [file pgen.1004811.s009.docx]

Table S7: Primers used in this study

| **Name** | **Sequence from 5' end** | **Use** |
| --- | --- | --- |
| LRL29 | tgtaaaacgacggccagtgccaagcttttggctgtgccctgcagctttc | Clone pLR6 |
| LRL30 | tacccggggatcctctagagtcgacgttggcgacgtttttcttggctttg | Clone pLR6, Test for deletion3 |
| LRL76 | ggttcccggaaactaagagc | Test for MAI |
| LRL78 | gcggttgtaggtttccttga | Test for MAI |
| LRL79 | atgacggaacgatcaagacc | Test for MAI |
| LRL103 | caaacgtctttggaggagga | Test for MAI |
| LRL128 | gctcggtacccggggatcctctagagctatctggggcggcattat | Clone pLR56 |
| LRL131 | caaagccaagaaaaacgtcgccaacgatgaaatacaaggcatgtgaaaag | Clone pLR20 |
| LRL132 | gctcggtacccggggatcctctagaggcgcatgcagaaatcttgt | Clone pLR20 |
| LRL139 | acgcctatcaatcgctgttc | Test for deletion2 |
| LRL141 | aactcggggtcctggtaaac | Test for deletion2 |
| LRL159 | caaagccaagaaaaacgtcgccaacgatggtcggggactttctgacg | Clone pLR41 |
| LRL160 | gctcggtacccggggatcctctagagggtcttcaagggcttgtgtc | Clone pLR41 |
| LRL161 | gtgcccaatcatcgaagg | Test for deletion1 |
| LRL162 | atcgtacgcgcttttacagg | Test for deletion1 |
| LRL177 | gccaagcttgcatgcctgcaggtcgaggcattgcagtgaagatgag | Clone pLR56 |
| LRL186 | aatttcacacaggaaacagaccatggtcggggactttctgacg | Clone pLR50 |
| LRL187 | tcagtggtggtggtggtggtgctcgaggcctccgcggacttccatga | Clone pLR50 |
| LRL190 | aatttcacacaggaaacagaccatgggcactgataataagcaatcattgc | Clone pLR52 |
| LRL191 | tcagtggtggtggtggtggtgctcgaggatttcgacctgagtacccagtt | Clone pLR52 |
| LRL204 | aatttcacacaggaaacagaccatgggtaaaggagaagaacttttcactg | Clone pLR58 |
| LRL205 | tcagtggtggtggtggtggtgctcgagtttgtatagttcatccatgccatg | Clone pLR58 |
| LRL207 | gctcggtacccggggatcctctagaggcaatcctgacagacttttcg | Clone pLR67 |
| LRL208 | caaagccaagaaaaacgtcgccaacgatgcagcttcccagtttcaggg | Clone pLR60 |
| LRL209 | gctcggtacccggggatcctctagagtgcaaacatccgctttgg | Clone pLR60 |
| LRL210 | caaagccaagaaaaacgtcgccaacgatgtcagatacttttgaagaatta | Clone pLR61 |
| LRL211 | gctcggtacccggggatcctctagagcagacaatccgccatgct | Clone pLR61 |
| LRL212 | caaagccaagaaaaacgtcgccaacgatgaaacagtttgaacaagatttc | Clone pLR62 |
| LRL213 | gctcggtacccggggatcctctagagactctccggccgtgctac | Clone pLR62 |
| LRL214 | caaagccaagaaaaacgtcgccaacgatgacaacggaatcgtatctgcac | Clone pLR63 |
| LRL215 | gctcggtacccggggatcctctagagattcccgacgtcagagttgt | Clone pLR63 |
| LRL217 | gctcggtacccggggatcctctagagtgcagatacgattccgttgt | Clone pLR68 |
| LRL218 | caaagccaagaaaaacgtcgccaacgatgcggcctgccgcgtcgtt | Clone pLR65 |
| LRL220 | gctcggtacccggggatcctctagagaccgccaaaccagacaataa | Clone pLR65 |
| LRL224 | gccaagcttgcatgcctgcaggtcgaagccgctgatctaccatcc | Clone pLR67 |
| LRL225 | gccaagcttgcatgcctgcaggtcgaacattgccaccatggatttt | Clone pLR68 |
| LRL231 | ccggacagaaatgcgaatgaatatatttataacaatatcatct | Test for deletion3 |
